# Enhancing iron biogeochemical cycling for *canga* ecosystem restoration: insights from microbial stimuli

**DOI:** 10.3389/fmicb.2024.1352792

**Published:** 2024-05-17

**Authors:** Rayara do Socorro Souza da Silva, Aline Figueiredo Cardoso, Rômulo Simões Angelica, José Augusto P. Bitencourt, Julio Cezar Fornazier Moreira, Adriano Reis Lucheta, Isabelle Gonçalves de Oliveira Prado, Dalber Ruben Sanchez Candela, Markus Gastauer

**Affiliations:** ^1^Instituto SENAI de Inovação em Tecnologias Minerais, Belém, Brazil; ^2^Instituto de Geociências, Universidade Federal do Pará, Belém, Brazil; ^3^Instituto Tecnológico Vale, Belém, Brazil; ^4^Instituto de Física, Universidade Federal Fluminense, Rio de Janeiro, Brazil

**Keywords:** duricrust, biocementation, microfossils, iron minerals, sustainable mining, Serra dos Carajás

## Abstract

**Introduction:**

The microbial-induced restoration of ferruginous crusts (*canga*), which partially cover iron deposits and host unique ecosystems, is a promising alternative for reducing the environmental impacts of the iron mining industry.

**Methods:**

To investigate the potential of microbial action to accelerate the reduction and oxidation of iron in substrates rich in hematite and goethite, four different microbial treatments (water only as a control − W; culture medium only − MO; medium + microbial consortium − MI; medium + microbial consortium + soluble iron − MIC) were periodically applied to induce iron dissolution and subsequent precipitation. Except for W, all the treatments resulted in the formation of biocemented blocks.

**Results:**

MO and MI treatments resulted in significant goethite dissolution, followed by precipitation of iron oxyhydroxides and an iron sulfate phase, due to iron oxidation, in addition to the preservation of microfossils. In the MIC treatment, biofilms were identified, but with few mineralogical changes in the iron-rich particles, indicating less iron cycling compared to the MO or MI treatment. Regarding microbial diversity, iron-reducing families, such as Enterobacteriaceae, were found in all microbially treated substrates.

**Discussion:**

However, the presence of Bacillaceae indicates the importance of fermentative bacteria in accelerating the dissolution of iron minerals. The acceleration of iron cycling was also promoted by microorganisms that couple nitrate reduction with Fe(II) oxidation. These findings demonstrate a sustainable and streamlined opportunity for restoration in mining areas.

## Highlights

- Iron-rich particles aggregate in response to different microbial stimuli by forming biofilms.
- Microbial action alters the levels of iron oxyhydroxides and results in the formation of biominerals.
- The growth of biofilms between grains consolidates *canga* fragments.
- Cellular structures provide active sites for mineral nucleation.
- Microorganisms with fermentative and respiratory metabolisms concomitantly contribute to the formation of biocements.

## 1 Introduction

Some of the largest iron ore deposits in the world are covered by ferruginous crusts generally outcropping on mountaintops, such as those in the Iron Quadrangle and the Serra dos Carajás, Brazil, or the Province of Hamersley, Australia (Dorr, 1964; Hagemann et al., 2016). These ferruginous crusts, also known as duricrust, *canga*, ferricrete, or ironstone, consist of a variety of materials bound together by iron oxyhydroxides, making them resistant to erosion (Dorr, 1964; Freyssinet et al., 2005; Shuster et al., 2012). Unique, ancient, and mega-diverse savanna-type ecosystems, containing dozens of microendemic species (Giulietti et al., 2019), have developed under the restricted environmental conditions provided by *canga* (Gibson et al., 2010; Jacobi and do Carmo, 2011). To reduce the impacts of mining on biodiversity and ecosystem services and make iron mining more sustainable, effective methods for *canga* duricrust restoration are needed (Gastauer et al., 2019).

Geochemical and geochronological evidence suggests that the ferruginous crusts from the Iron Quadrangle and Serra dos Carajás were formed through repeated cycles of iron mineral dissolution and reprecipitation triggered by, among other factors, iron-reducing microorganisms (biocementation). Goethite formation within *cangas* ranges from ~80 Ma to 1 Ma, with younger ages closer to the surface and progressively older ages toward the saprolite, indicating the importance of biological processes for iron cycling (Monteiro et al., 2014, 2018; Gagen et al., 2019a). Near the surface, locally anaerobic conditions facilitate the dissolution of iron oxyhydroxides through iron reduction induced by microorganisms. During this process, Fe(III) serves as an electron acceptor in microbial respiration, oxidizing hydrogen or carbon compounds (Lovley, 1991; Kappler et al., 2021; Dong et al., 2023). As the environment shifts toward aerobic conditions, Fe(II) oxidizes back to ferric iron and precipitates, generating biocements (Zammit et al., 2015). Although the natural cycling of iron minerals in ferruginous crusts has developed over millions of years, laboratory experiments have demonstrated that this process can be accelerated by microbial activity (Gagen et al., 2020; Levett et al., 2020a).

Recent studies have identified the presence of iron-reducing bacteria in Serra dos Carajás across various environments, suggesting their widespread distribution (Gagen et al., 2020; Cardoso et al., 2023). The cultivation of these microorganisms can be promising for *canga* restoration, thereby fostering sustainable mining. Here, we aimed to examine the mineralogical alterations in an iron-rich substrate subjected to repeated cycles of irrigation and desiccation in the laboratory using three different microbial stimuli and a control treatment (W: water as a control, MO: culture medium only, MI: culture medium + iron reducing microbial consortium, MIC: culture medium + iron reducing microbial consortium + dissolved iron). We examined alterations in substrate texture using stereomicroscopy and scanning electron microscopy and carried out chemical analysis of the substrates from the distinct treatments. Furthermore, we used XRD and Mössbauer spectroscopy to track alterations in mineralogical composition. Additionally, we analyzed the composition of the iron-reducing microbial consortium, as well as the composition of the bacterial communities in the iron-rich substrate. We expected that the stimulation of microorganisms together with repeated cycles of dissolution and subsequent precipitation of iron in all treatments except W would trigger alterations of the substrates and lead to their cementation and cohesion.

## 2 Materials and methods

### 2.1 Experimental setup

To induce biocementation under controlled conditions, we irrigated an iron-rich substrate three times per week to simulate short cycles of waterlogging (anaerobic conditions for iron reduction) and desiccation (aerobic conditions for iron precipitation) for 5 months. We used a control treatment (W: water only) and three different microbial stimuli (MO: culture medium only, MI: culture medium + iron-reducing microbial consortium, MIC: culture medium + consortium + dissolved iron). All treatments were tested in triplicate; replicates were placed in an incubator (Innova^®^ 42) throughout the experiment at 28°C (Supplementary Figure S1).

The culture media for the MO, MI, and MIC treatments were DSMZ 579 (2.5 gL^−1^ NaHCO_3_; 1.5 gL^−1^ NH_4_Cl; 0.60 gL^−1^ NaH_2_PO_4_; 0.1 gL^−1^ KCl), supplemented with 10 mL.L^−1^ of each vitamin and micronutrient [DSMZ 141: 10 gL^−1^ ZnSO_4_.7H_2_O; 1.0 gL^−1^ CuSO_4_.5H_2_O; 1.0 gL^−1^ MnSO_4_.4H_2_O; 1.0 gL^−1^ CoSO_4_.7H_2_O; 0.5 gL^−1^ Cr_2_(SO_4_)_3_.15H_2_O; 0.6 gL^−1^ H_3_BO_3_; 0.5 gL^−1^ Na_2_MoO_4_.2H_2_O; 1.0 gL^−1^ NiSO_4_.6H_2_O; 1.0 gL^−1^ Na_2_SeO_4_.10H_2_O; 0.1 gL^−1^ Na_2_WO_4_.2H_2_O; 0.1 gL^−1^ NaVO_3_]. The culture media was sterilized by autoclaving for 15 min at 121°C. The MO and MI treatments were supplemented with a glucose solution (10 mM), while the MIC was enriched with Fe(III) citrate (50 mM) and Na-acetate (30 mM). The pH of the solutions was adjusted to 6.8 using 1 M H_2_SO_4_, and oxygen was removed to maintain anaerobic conditions by flushing N_2_ (filtered through Millex^®^ filter 0.22 μm) into the system with a purge. All treatments were set up using carefully sealed 1 L Schott bottles.

The microbial consortia utilized in both the MI and MIC treatments consisted of a blend of 4-SS and 6-SN consortia, which were previously chosen for their iron-reducing capabilities (Cardoso et al., 2023). Each treatment was inoculated with 50 mL of each consortium, with a starting concentration of at least 2×10^5^ CFU mL^−1^. The total volume was adjusted to 1 L with the culture medium described above. These microbial cultures were then incubated in a shaker chamber at a constant temperature of 28°C and 163 rpm throughout the experiment.

The concentration of dissolved Fe(II) from the MI and MIC cultures was measured monthly by spectrophotometry using the modified ferrozine assay of Lovley and Phillips (1986). For evaluation, 0.1 mL samples were taken in triplicate from the microbial cultures and acidified with 5 mL of 0.5 M HCl. Additionally, a control sample consisting of the same culture medium but without the microbial consortium was prepared following the same protocol. After allowing the samples to rest for 15 min, 0.2 mL of the mixture was homogenized with 2 mL of ferrozine (1 gL^−1^ in 50 mM HEPES) and filtered through a filter with a pore size of 0.2 μm. The absorbance was measured at 562 nm and compared with that of known standard concentrations of Fe(II) prepared with ferrous sulfate. In the MIC culture, the Fe(II) concentration oscillated between 2 and 3 mM, while the dissolved Fe(II) in the MI culture was below detection limit throughout the experiment (Supplementary Figure S1).

For biocementation, crushed ferruginous crusts from one of the Serra Norte mining fronts were selected as substrates. Prior to the experiment, an XRD analysis (for methodological details, see section 2.2) of the substrate indicated the presence of hematite, goethite, gibbsite, and anatase in its composition (Supplementary Figure S2). Prior to the experiment, the substrate was sieved with an electromechanical shaker to withdraw particles larger than 4 mm. Subsequently, approximately 8.4 kg of this fraction was split in equal parts into 12 plastic containers (3 cm × 11 cm × 11 cm).

Each plastic container received 15 mL of one of the solutions described above per application (3x per week); this corresponds to a precipitation of 1.3 Lm^−2^ per application. This process was sufficient to humidify the substrate and create local anaerobic conditions to trigger iron reduction. After 5 months of periodic irrigation, the boxes remained for 1 month without receiving irrigation, aiming at dehydration for the final consolidation of the substrates. After this period, samples were collected for subsequent analyses.

### 2.2 X-ray diffraction (XRD)

To quantify the mineralogical changes of the substrates in each treatment, analysis was performed by X-ray diffraction (XRD) using the powder method and backloading preparation. The analysis was performed with a PANalytical Empyrean diffractometer (CoTube, 1 = 1.78901 Å; Long Fine Focus, Fe Kβ Filter, PIXcel3D-Medpix3 1×1 detector) in scanning mode with a voltage of 40 kV and current of 30 mA. For the Rietveld refinement, the following parameters were used: step size of 0.0130° in 2θ, scan from 4° to 109° in 2θ, time/step of 68.6 s, divergent slit: 1/8° and anti-scattering: 1/4°, and mask: 10 mm. The mineral phases were identified and quantified using the International Center for Diffraction Data (ICDD) database with X’Pert HighScore 3.0 software.

### 2.3 Mössbauer spectroscopy

^57^Fe Mössbauer spectroscopy was performed to quantify the major iron-bearing phases (>1%) in the transmission geometry. For that, the samples were kept at 4 K inside a close cycle Janis cryostat with the Co-57 source (in the Rh-matrix) moving sinusoidally at room temperature. All the isomer shift IS values were obtained relative to those of metallic iron. These tests were performed in the laboratory of the Physics Institute of the Fluminense Federal University.

### 2.4 Microtextural analysis

Petrographic sections from consolidated samples (treatments MO, MI, and MIC), polished to a thickness of 30 μm, were embedded in Araldite resin. Sections were analyzed under a Zeiss SteREO Discovery V12 stereomicroscope coupled with a Zeiss Axiocam 712 camera (12 megapixels). These samples were then analyzed with a scanning electron microscope (Zeiss, Sigma-VP) equipped with energy dispersion X-ray spectroscopy (EDS, IXRF Sedona-SD) at an acceleration voltage of 20 kV.

To image biofilms after the experiment, millimetric pieces of consolidated and unconsolidated samples from all treatments were fixed in 2.5% glutaraldehyde with phosphate buffer solution (PBS) for at least 2 h, followed by rinsing with PBS. Dehydration was carried out with different concentrations of ethyl alcohol (50, 60, 70, 80, 90%, and 2 × 100%), with subsequent drying at the critical point (Leica CPD 300). To improve the electrical conductivity, all the samples were previously coated with 15 nm of gold using an Emitech K550X sputter coater. Measurements of the cell structures in the SEM images were carried out with ImageJ software.

### 2.5 Whole-substrate chemistry

Chemical analysis of the total sample to determine the major, minor, and trace elements, including rare earth elements, was performed by SGS Laboratories. To investigate the chemical behavior under different stimuli. The major elements (SiO_2_, Al_2_O_3_, Fe_2_O_3_, CaO, MgO, Na_2_O, K_2_O, TiO_2_, MnO, and P_2_O_5_) were determined by X-ray Fluorescence (XRF), after fusion of the samples with lithium tetraborate (SGS analytical code XRF79C). The loss on ignition (LOI) was obtained by calcination at 1000°C (SGS analytical code PHY01E). The concentrations of trace elements (Be, Sc, V, Cr, Co, Cu, Zn, Ga, Ge, As, Rb, Y, Zr, Nb, Mo, Ag, Cd, In, Sb, Cs, Ba, Hf, W, Hg, Pb, Bi, Th, and U) were determined via inductively coupled plasma–mass spectrometry (ICP–MS) and inductively coupled plasma–optical emission spectrometry (ICP–OES) after digestion by aqua regia (SGS analytical code ICM14B). Other elements, such as Ni and rare earth elements (La, Ce, Pr, Nd, Sm, Eu, Gd, Tb, Dy, Ho, Er, Tm, Yb, and Lu), were analyzed via ICP–MS. Other parameters, such as total carbon and sulfur content, were obtained using a LECO Carbon Sulphur Analyzer (SGS analytical code CSA17V).

### 2.6 DNA extraction, sequencing, and sequence analysis

DNA extraction was carried out from the two microbial cultures utilized and from 12 substrate samples (three from each treatment). For the microbial cultures, a volume of 15 mL was filtered through 0.22 μm sterile filter membranes (Millipore, Merck KGaA, Darmstadt, Germany). Subsequently, these membranes were manually cut into small pieces with a sterile scalpel. Approximately 300 mg of each substrate sample was collected for analysis after the completion of the experiment.

For both the microbial cultures and substrates, DNA extraction was performed using a PowerSoil DNA Isolation Kit (QIAGEN) according to the manufacturer’s instructions. DNA samples were quantified using a Qubit 3.0 fluorometer (Thermo Fisher Scientific, Inc.), and the DNA quality was checked via electrophoresis on a 1% agarose gel.

Bacterial diversity was evaluated by sequencing the V3–V4 region of the 16S rRNA gene using the Illumina MiSeq platform. For this purpose, approximately 5 ng μL^−1^ extracted DNA was subjected to PCR. The gene-specific sequences used targeted the V3–V4 region of the 16S rRNA gene (Klindworth et al., 2013). Illumina adapter overhang nucleotide sequences were added, and this region was amplified using the full-length primer set 16S Forward Primer = 5′ TCGTCGGCAGCGTCAGATGTGTATAAGAGACAGCCTACGGGNGGCWGCAG 3′ and 16S Reverse Primer = 5′ GTCTCGTGGGCTCGGAGATGTGTAAGAGACAGGACTACHVGGGTATCTAATCC 3′. The PCR mixture was combined with ultrapure water to prepare a final volume of 25 μL. The mixture contained 1.25 μL of dNTPs (2 mM), 2.0 μL of MgCl_2_ (25 mM), 0.5 μL of each primer (10 μM), 1 μL of DNA (5 ng), 1 μL of Platinum™ Taq DNA polymerase (0.1 U) (Invitrogen, Waltham, MA, United States), and 5 μL of 10× Buffer. Amplification was performed in an Applied Biosystems thermocycler with an initial cycle of 3 min at 95°C; 25 cycles of 30 s at 95°C, 30 s at 57°C, and 30 s at 72°C; and a final cycle of 5 min at 72°C. The amplicons were quantified using a Qubit 3.0 fluorometer (Thermo Fisher Scientific, Inc.), and the quality was measured on a 1% agarose gel.

Then, the amplicon libraries for the bacteria were prepared according to the Illumina 16S Metagenomic Sequencing Library Preparation Protocol (Illumina, San Diego, CA, United States). The PCR products were prepared in a final volume of 25 μL. The PCR mixture contained 12.5 μL of 2x Kappa Hifi HotStart Ready Mix (Sigma–Aldrich, St. Louis, MI, United States), 10 μL of Index primer, 2.5 μL of DNA, and 5 μL of PCR water. The PCR cycle for bacteria consisted of an initial denaturation of 3 min at 95°C, followed by 8 cycles of denaturation at 95°C for 30 s, annealing at 57°C for 30 s, extension to 72°C for 30 s, and a final extension to 72°C for 5 min.

The size and quality of the PCR fragments were estimated on an Agilent 2100 Bioanalyzer (Agilent Technologies, Santa Clara, CA, United States) using a DNA 1000 chip. The PCR products were purified with an AMPure XP purification kit (Beckman Coulter, Brea, CA, United States), and the libraries were further processed with a Nextera XT kit (Illumina). The libraries were standardized to a concentration of 4 nM and processed following Illumina 16S Metagenomic Sequencing Library Preparation (Illumina). The 16S rDNA gene libraries were sequenced on the MiSeq-Illumina platform using the MiSeq V3 reagent kit (600 cycles; Illumina) at Instituto Tecnológico Vale – ITV-DS (Belém, PA, Brazil).

Bioinformatic analysis was performed using Quantitative Insights Into Microbial Ecology Software (QIIME) according to the Pipeline for MetaBarcoding Analysis (PIMBA) (Oliveira et al., 2021) for 16S rRNA sequences. The sequences were trimmed and filtered by quality and converted to FASTA using Prinseq v0.20.4. VSEARCH v2.15.2 was used to dereplicate and discard singletons, trim reads, and group reads into operational taxonomic units (OTUs) with 97% similarity and remove chimeras. Taxonomic assignment was performed using the ribosomal RNA database (Quast et al., 2013). After that, the data were filtered in R software, and OTUs that were not categorized as bacteria were removed. For functional characterization of the detected taxa, we applied the Functional Annotation of Prokaryotic Taxa prediction tool (FAPROTAX, version 1.2.4). The FAPROTAX database maps the metabolic functions of bacterial OTUs into putative functional profiles, enabling the ecological interpretation of 16S marker gene data. The analysis was performed using the collapse_table.py script (Louca et al., 2016).

## 3 Results

### 3.1 Consolidation and mineralogy of *canga*

**Figure 1:**
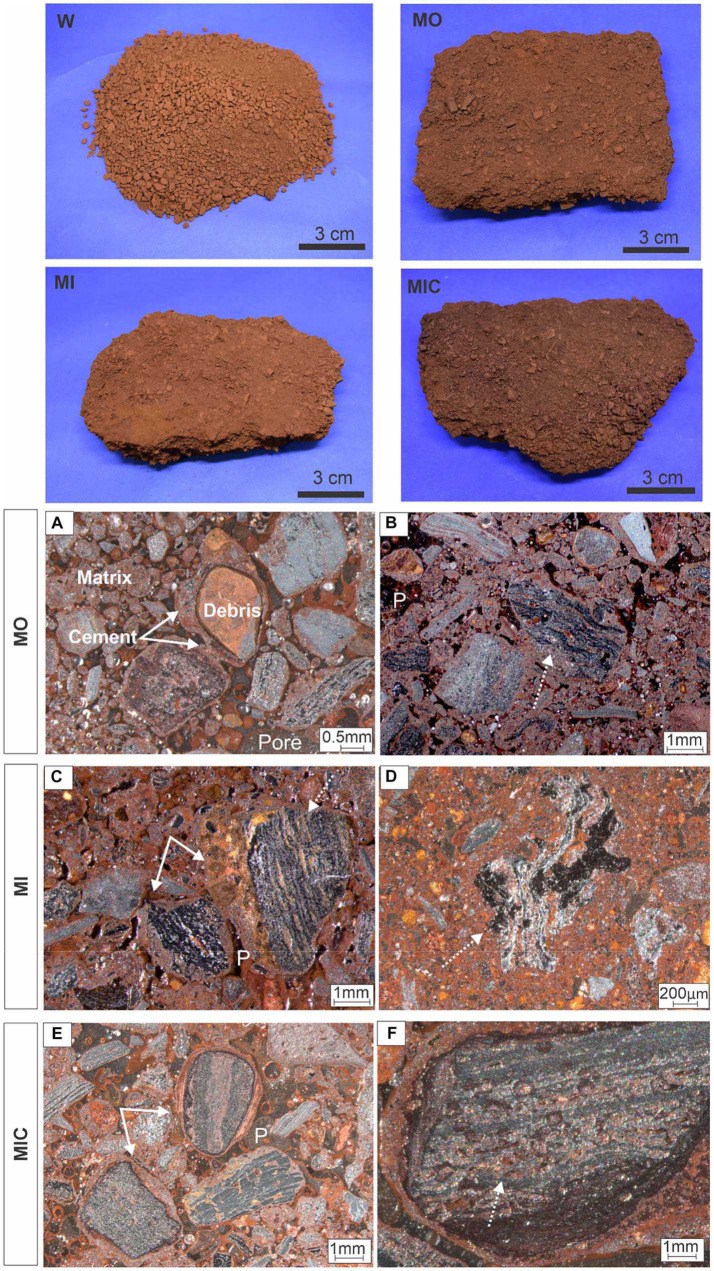
Consolidation of *canga* fragments after 6 months of experimentation (upper block) and textural aspects under different biocementation conditions. Hematitic debris (>2 mm) interconnected by the newly formed cemented material **(A)** and with dissolution along the relict structures **(B-D)**. Cement overgrown over hematite debris **(E)**. Secondary porosity of the cellular molds **(F)**.

The experiments with unconsolidated *canga* resulted in different degrees of response to each applied treatment, as follows. The substrate of treatment W remained unconsolidated, with no evidence of biocementation. Conversely, treatments MO, MI, and MIC led to the formation of cohesive and compact ferruginous samples (Figure 1). The obtained blocks have a detrital aspect containing clasts composed mostly of hematite, followed by iron oxyhydroxide (hematite and goethite) and minor amounts of gibbsite (Table 1).

**Table 1:** Mineral phases quantified by Rietveld refinement for each treatment and their respective Rietveld indices (GOF is the goodness-of-fit, and R_WP_ is the weighted profile parameter) for the treatment: water (W, control), culture medium only (MO), medium + microbial consortium (MI), and medium + microbial consortium + soluble iron (MIC).

Phases	W	MO	MI	MIC
Hematite (%)	64.1	67.8	65.6	63.8
Goethite (%)	32.9	30	30.7	34.2
Gibbsite (%)	2.8	2.1	3.4	1.8
Anatase (%)	0.3	0.1	0.3	0.3
GOF	1.35	1.32	1.30	1.26
*R*_WP_	5	4.89	4.84	5

In microtextural terms, a matrix with fine debris (<1 mm) of iron oxyhydroxide surrounded by an authigenic cement that adheres to other debris of different size proportions was common among the consolidated treatments (Figure 1). Some of these debris exhibited irregular edges indicative of dissolution, filled with iron-aluminous cements.

Hematite clasts with diameters greater than 2 mm are evidenced by dissolution features, preferably along relict banding (Figures 1C,E), and are individualized by cement that indicates the continuity of these particles. Some clasts are even replaced by this recent cement, preserving only the mineral habit. The intraparticle porosities exhibited very regular shapes that resembled those of bacteriomorphs, demonstrating secondary porosity (Figure 1E).

Gibbsite crystals are also frequent along the cavities and at the edges of the cemented debris (Supplementary Figure S3). Occasionally, there are zones of Fe oxyhydroxide intercalated with gibbsite, indicating dissolution and reprecipitation of both minerals during the process.

The mineralogical composition of the MO, MI, and MIC treatments differed quantitatively from that of the W treatment (control) (Table 1). The concentrations of goethite decreased, while those of hematite increased in the MO and MI treatments, unlike in the MIC treatment, in which there was an increase in goethite. The gibbsite also varies, with higher concentrations in the MI and lower concentrations in the MIC. Anatase exhibited almost no changes.

**Figure 2:**
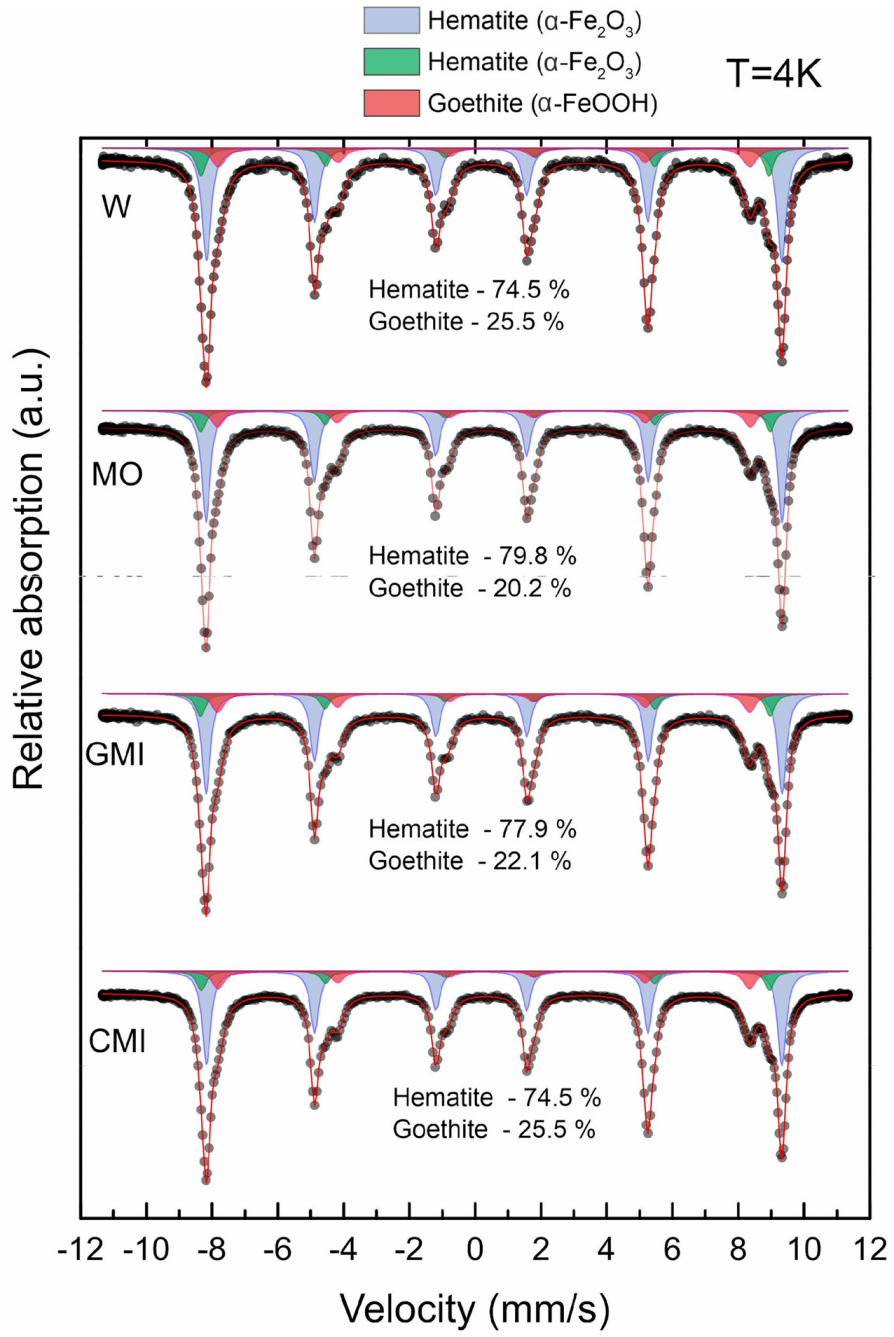
Distribution of iron minerals in samples from each treatment, according to Mössbauer spectra at 4 K. W, water (control); MO, culture medium; MI, culture medium + iron reducing microbial consortium; MIC, culture medium + consortium + dissolved iron.

The concentrations of the iron phases detected by Mössbauer spectroscopy (>1%) (Figure 2) varied only between the MO and MI treatments compared to W, with the decrease in goethite being greater in the MO treatment.

The low-temperature Mössbauer spectroscopy data indicate that the samples from all the treatments produced two sextets, corresponding to hematite, and one sextet, corresponding to goethite. However, hyperfine magnetic field values varied according to treatment (Supplementary Table S1).

### 3.2 Biofilm formation

**Figure 3:**
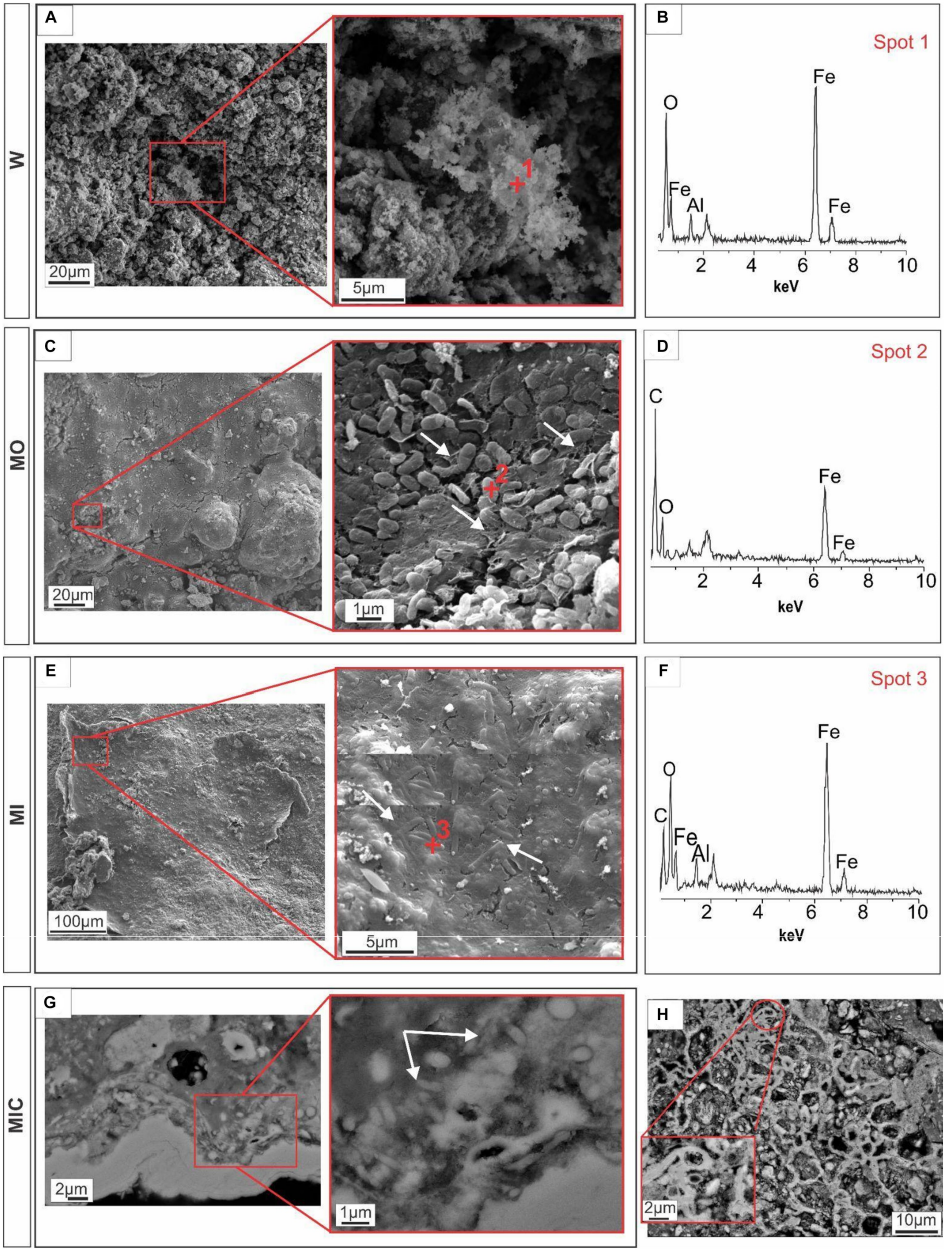
Microtexture (SEM) and chemical composition (EDS) of biofilms produced during the experiment. W, control: Control group, displaying iron-rich loose particles **(A,B)**. MO treatment: compact surface of EPS (extracellular polymeric substances) with iron oxyhydroxides, partly embedding rod-shaped cells **(C),** and respective EDS spectrum **(D)**. MI treatment: Filamentous cells embedded in EPS and iron oxyhydroxides **(E)** and the corresponding EDS spectrum **(F)**. MIC treatment: Biofilms in the cavity; details of the cell envelopes are shown (arrows) **(G)**; and biofilms with preserved cellular structures are shown **(H)**.

Scanning electron microscopy (SEM) revealed biofilm formation under the different conditions during which the biogeochemical cycle of Fe was stimulated, except for treatment W. Unlike all other treatments, the iron-rich particles in treatment W remained loose, with rough relief (Figures 3A,B). In the MO and MI treatments, sets of well-preserved cells were observed coating the Fe oxyhydroxide particles. Some of these sets of cells appear to be bound to extracellular polymeric substances (EPS), which aid in the attachment of cells to Fe oxyhydroxide surfaces, resulting in smooth relief. The presence of cells is clearer in Figure 3C, where one can see fossilized cells with an average size of 0.8 μm in the form of rods, partially embedded in EPS; and in Figure 3E, with 0.3 μm cell filaments, completely embedded in EPS. EDS revealed the presence of carbon and iron associated with fossilized cells (Figures 3D,F).

**Figure 4:**
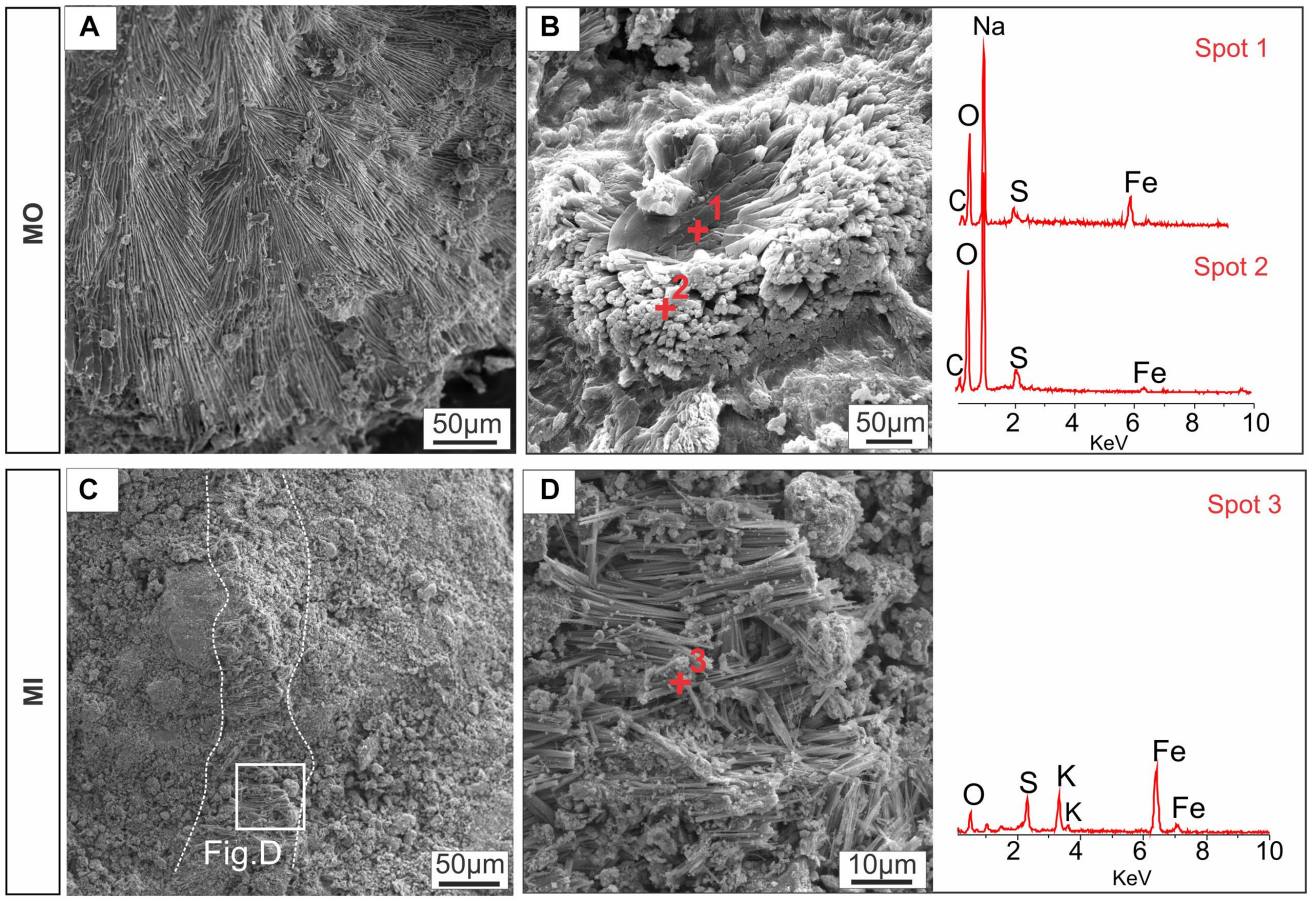
Textural aspects of the iron-bearing sulfate compounds detected by SEM–EDS: sodium-iron-bearing sulfate compounds with fibroradial habits **(A)** and nucleation of elongated structures **(B)**; and potassium-iron-bearing sulfate along a vein feature **(C)** and with a fibrous appearance **(D)**. MO, culture medium only; MI, medium + microbial consortium.

Iron-bearing sulfate compounds (with sodium and potassium) were detected by EDS in the biofilms from the MO and MI treatments. The sodium-rich variety exhibited a fibrous set with a radial arrangement (Figures 4A,B). Conversely, the potassium-rich variety occurs as fibrous veins (Figures 4C,D) and small fragments dispersed in the cement.

**Figure 5:**
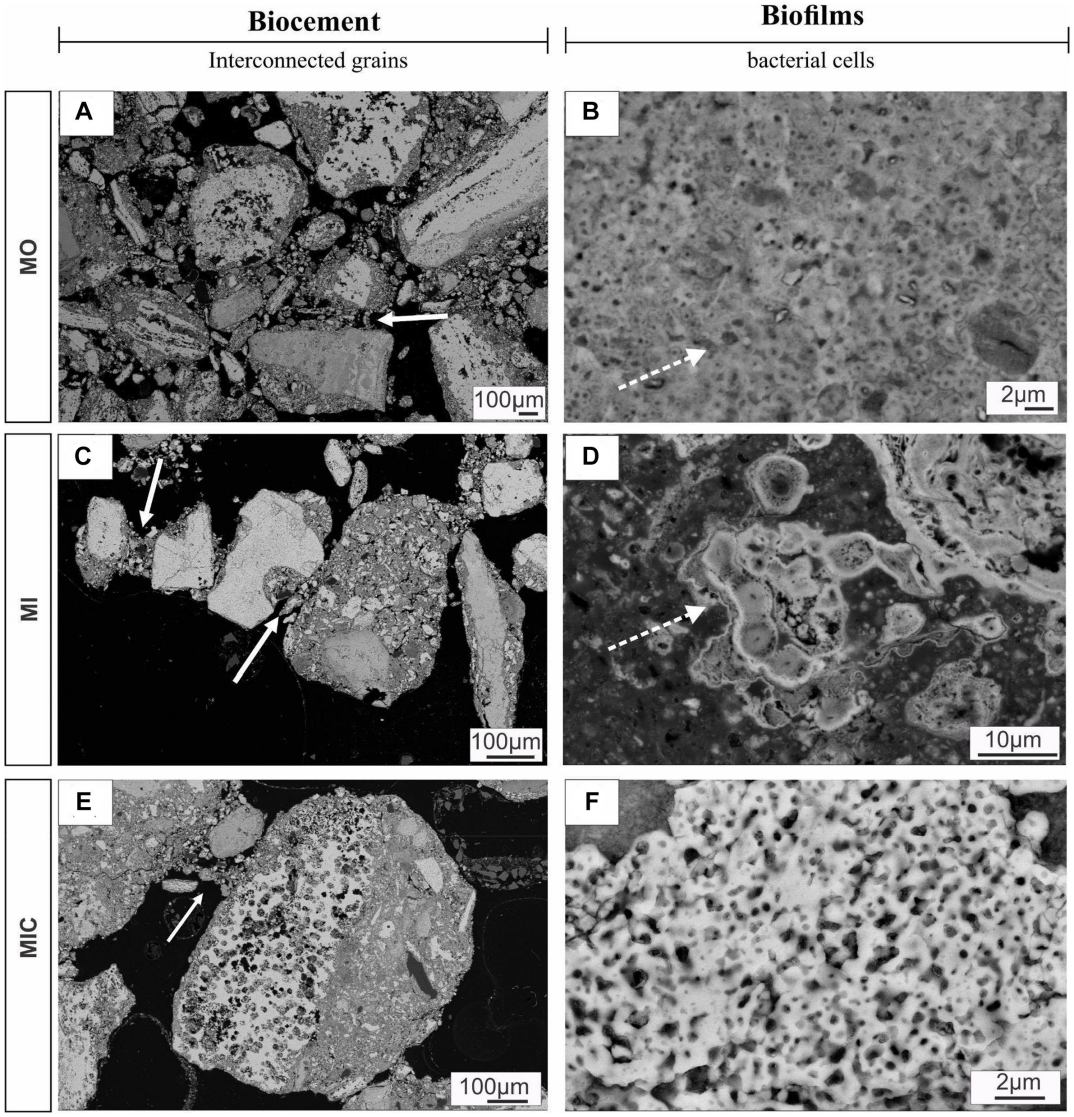
SEM images of polished sections displaying biocements and biofilms. Panels **(A,C,E)** show different debris layers interconnected by meniscus-type cements (solid arrows), while **(B,D)** indicate the nucleation of iron oxyhydroxides on the surface of cell envelopes (solid arrows). Panel **(F)** illustrates a set of bacteriomorphs in MIC treatment. Culture medium only (MO), medium + microbial consortium (MI), and medium + microbial consortium + soluble iron (MIC).

In the MIC treatment, iron-bearing sulfate compounds were not detected, and Fe-oxyhydroxide precipitates filled part of the cavities associated with biofilm structures, which comprised rods and filament-shaped cells (Figure 3G). In general, after Fe(III) citrate treatment (MIC), the microbial cells in the biofilms were more likely to be bacteriomorphic molds with little cell envelope structure, as shown via the polished section (Figure 3H). On the hematite surface, several pores, left by cellular structures reaching 0.3 μm in diameter, are frequent (Figure 5F), features also visible in the glucose-enriched substrate samples (MO and MI).

In addition to the cellular structures, another aspect observed in all the treatments (except for W) was the formation of meniscus-like structures that interconnected the substrate particles (Figures 5A,C,E), forming microaggregates. At the edge of the particles (cement), the cell envelopes frequently formed nucleation surfaces for the precipitation of iron oxyhydroxides (Figures 5B,D).

### 3.3 Chemical composition of the substrates

**Figure 6:**
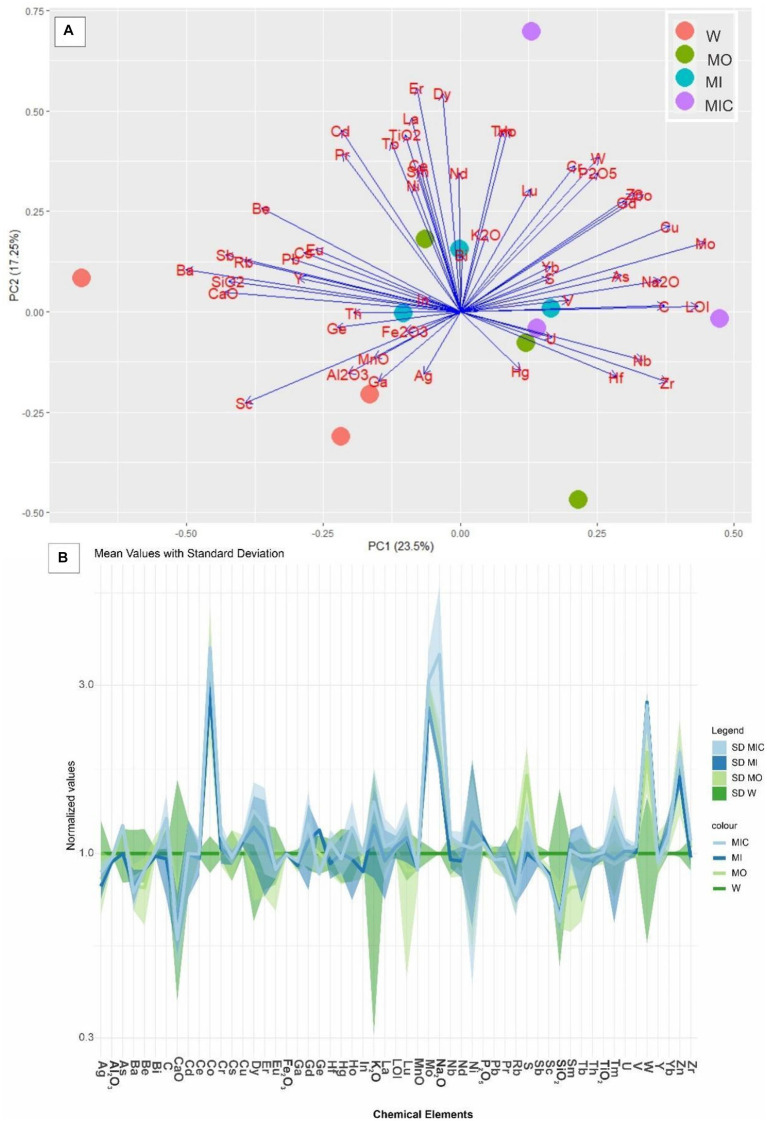
**(A)** Principal component analysis (PCA) of the total chemical composition of the substrates of the different treatments. **(B)** Distribution pattern of the concentrations of the trace elements in the treatment groups normalized to those in the control group. Treatments: Water (W, as a control), culture medium only (MO), medium + microbial consortium (MI), and medium + microbial consortium + soluble iron (MIC).

In the substrates of all treatments, Fe_2_O_3_ was the most abundant oxide (90–90.5%), followed by Al_2_O_3_ (2.9–3%), TiO_2_ (0.3–0.4%), SiO_2_ (0.4–0.7%) and P_2_O_5_ (0.3–0.4%, Supplementary Tables S2 and S3). The concentrations of other oxides were lower than 0.1%, which is the detection limit. Despite this similarity, there was a difference between the control treatment and the other treatments (*p* = 0.011), as highlighted in Figure 6A. The trace element concentrations indicate that the main differences in the concentrations of C, S, Co, Cu, Zn, Mo, Cr, and W occur at ratios between 1 and 3 when these concentrations are normalized as a function of the W treatment (Figure 6B).

### 3.4 Microbial communities

**Figure 7:**
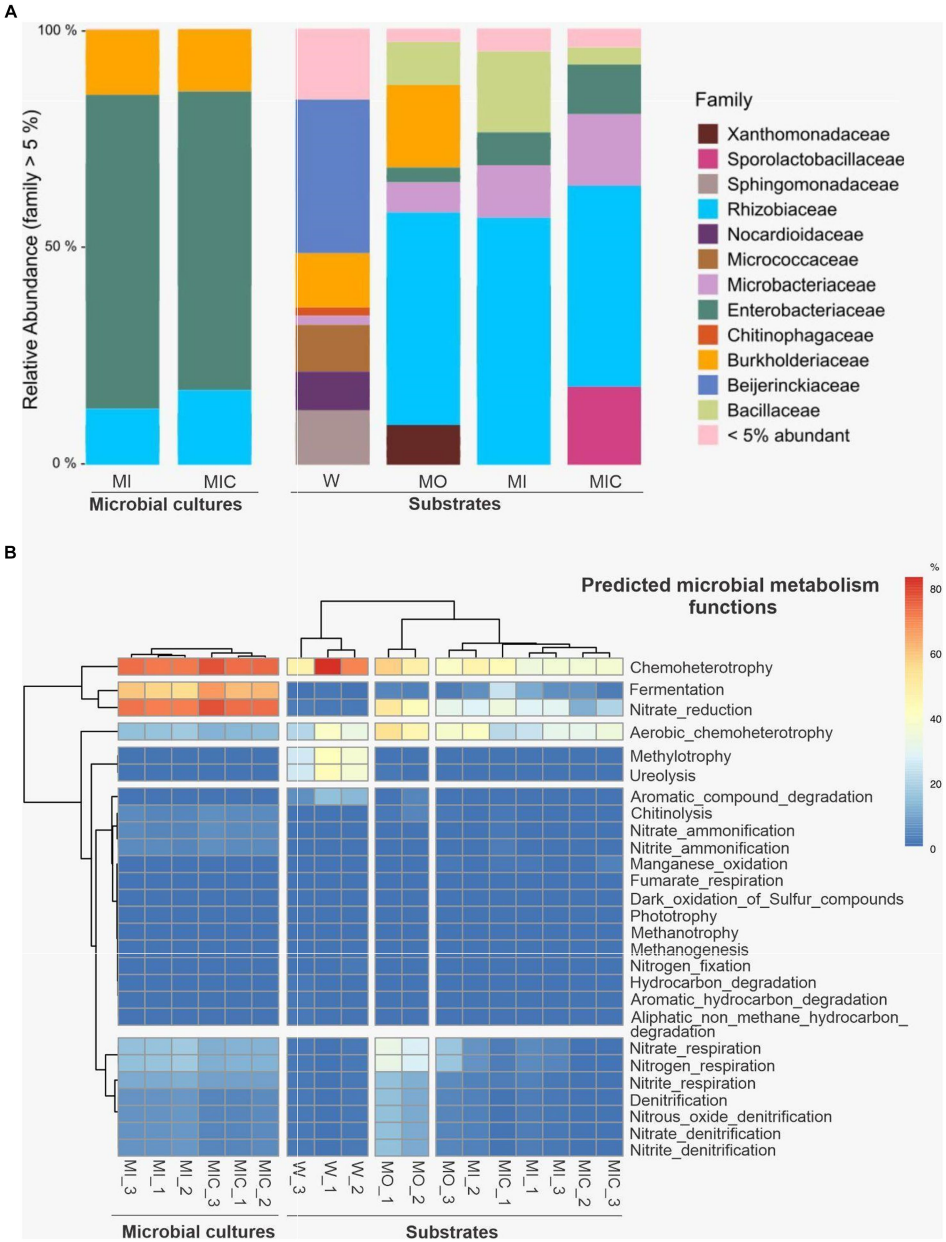
Illumina 16S rRNA sequence data were extracted from substrates and microbial cultures; only bacterial data were accessed. **(A)** Average relative abundance of microbial cultures and substrates at the family level. **(B)** Cluster analysis of predicted microbial metabolism functions of microbial cultures and substrates. Treatments: Water (W, as a control), culture medium only (MO), medium + microbial consortium (MI), and medium + microbial consortium + soluble iron (MIC).

A total of 681,662 sequences were obtained from the two microbial culture samples, and 393,259 sequences were recorded from the 12 substrate samples. After quality filtering and removal of sequences not linked to bacteria, we obtained 298 OTUs in the glucose microbial culture (MI) and 316 OTUs in the Fe(III) citrate microbial culture (MIC, Supplementary Table S4). In both microbial cultures, a predominance of OTUs classified into the families Rhizobiaceae, Enterobacteriaceae, and Burkholderiaceae was identified (Figure 7A).

Among the substrates, 240 OTUs were identified in the W treatment, 262 OTUs were identified in the MO treatment, 268 OTUs were identified in the MI treatment, and 187 OTUs were identified in the MIC treatment. The microbial community profiles of the samples collected from the microbial cultures and from the substrates at the end of the experiment were proportionally different. In the microbial culture samples, the Enterobacteriaceae family was predominant, and the family Rhizobiaceae was more abundant in the substrates.

The composition of the microbial communities was influenced by the treatment to which the substrate was subjected (Figure 7A). The treatment that includes only the culture medium comprised the Burkholderiacea family, which was also identified in the W treatment. In addition, the Xanthomonadaceae family was found only in the MO treatment, while the Sporolactobacillaceae family was identified exclusively in the MIC treatment. To a lesser extent, the Enterobacteriaceae, Microbacteriaceae, and Bacillaceae families were identified among the substrates of the MO, MI, and MIC treatments.

The predicted microbial metabolic functional groups based on 16S rDNA sequences indicated differences between microbial cultures and substrates (Figure 7B). In bacteria that grow in microbial cultures, the dominant metabolic pathways are related to anaerobic activity, with most organisms putatively performing activities related to chemotaxis and fermentation and, secondarily, pathways related to the cycling of ammonia, as well as nitrite and nitrate. For substrates, the predicted metabolic pathways of the control treatment (W) were less diverse and based on aerobic chemoheterotrophy, in addition to recycling of nitrogenous excreta (ureolysis) and carbon (methylotrophy). Conversely, in the substrates treated with glucose without a consortium (MO), the native microorganisms presented more evident nitrate reduction activities, in addition to aerobic chemoheterotrophy. In the substrates that received microbial culture (MI and MIC), the metabolic pathways were mainly related to chemoheterotrophy but had lower nitrate reduction activities in relation to MO.

## 4 Discussion

Within our experiment, various conditions were manipulated to stimulate the biogeochemical cycle of iron, ultimately leading to the formation of biocemented blocks. By supplying micro-and macronutrients, along with carbohydrates, and subjecting the system to repeated cycles of irrigation and subsequent desiccation, we induced alterations in the microbial community and triggered mineralogical and textural changes in *canga* fragments. Notably, nodules surrounded by authigenic cements were observed, with these nodules adhering to debris of varying sizes, likely stemming from the dissolution of iron minerals. The evidence of bacterial dissolution molds on the surface of these minerals further supported this hypothesis. Additionally, the presence of microfossils within the authigenic cements indicated their role in aggregating the enveloped particles, ultimately contributing to the formation of biocemented blocks within the tested systems.

### 4.1 Mineralogical and chemical modifications

Our experiments revealed substantial mineralogical changes within the treated substrates, reflecting the intensification of iron mineral dissolution-precipitation cycles compared to seasonal changes in natural *canga* environments (Monteiro et al., 2014; Levett et al., 2016, 2020b; Spier et al., 2018; Gagen et al., 2019a; Parker et al., 2022). These changes were revealed independently by two distinct methods, Mössbauer spectroscopy and Rietveld refinement. Values differ slightly between methods because Rietveld refinement quantifies all phases in the sample, while Mössbauer spectroscopy only quantifies iron-bearing phases. Additionally, Mössbauer results can be affected by crystal size, which affect the intensity of the hyperfine field, especially in cryptocrystalline materials. Nevertheless, both methods indicate the same tendencies in our analysis. While iron oxide phases differ between treatments, the amount of accessory anatase remained unchanged across all treatments, and gibbsite displayed point changes in all treatments, except for W, due to solubilization by microbial activity amid pH and organic chemistry changes (Levett et al., 2020b).

The manipulation of microbial stimuli and carbon sources significantly influenced the iron cycle, with notable goethite dissolution observed principally in treatments enriched with glucose (MO and MI). These treatments promoted the development of fermentative and anaerobic bacteria that supply hydrogen to the system during the breakdown of glucose and organic byproducts. Hydrogen served as an electron donor for direct iron reduction, facilitating iron mineral dissolution (particularly goethite), as previously demonstrated by Gagen et al. (2019b). Goethite is poorly crystalline and more soluble than more ordered minerals such as hematite and thus has a notably greater dissolution susceptibility (Cornell and Schwertmann, 2003; Bird et al., 2011).

In the MO and MI treatments, the resultant availability of Fe(II), along with its subsequent oxidation to Fe(III), likely precipitated newly formed minerals, including iron sulfates, amid acidic microenvironments promoted by glucose fermentation. The formation of sodium-and potassium-bearing iron sulfates may have been catalyzed by the decomposition of chemical compounds present in the culture medium (Nazari et al., 2014; Cruells and Roca, 2022), which is thus the result of biomineralization associated with iron-oxidating/reducing bacteria (Lazaroff, 1997), highlighting the multifaceted role of microbial communities in mineral cycling.

The reduction of Fe(III) from goethite and hematite by microbial cultures in the presence of acetate/Fe(III) citrate (MIC treatment) was lower than that in the glucose treatments (MO and MI). This finding is consistent with that observed by Lentini et al. (2012) and Castillo-Zacarías et al. (2020), who found that feeding microbes with alternative carbon source had observed low iron reduction compared to feeding them with glicose. Furthermore, the applied concentration of Fe(III) citrate (50 mM) in the MIC treatment may be toxic to studied microorganisms (Chen et al., 2013). Before introducing the microbial culture into the substrate, the bacteria interacted with Fe(III) citrate, resulting in the consumption of Fe(III) and the release of Fe(II). Prolonged exposure of cell surfaces to soluble Fe(II) may have inhibited their activity (e.g., Roden and Urrutia, 2002; Zhang et al., 2020).

The high contents of C, S, Co, Zn, Mo, Cr, W, and Cu in blocks from different treatments indicate that chemical components applied as nutrients were, at least partially, fixed as newly formed minerals or adsorbed by the precipitated iron oxyhydroxides (Weber et al., 2006; Alfadaly et al., 2021; Keim et al., 2021). Moreover, free-floating bacteria can produce siderophores, organic compounds known for their strong affinity for metals that function to chelate bioavailable iron(III) and various other metals from minerals (Gadd, 2004; Lies et al., 2005; Sheng et al., 2023).

### 4.2 Biocements

Our study revealed that fossilized bacteria acted as nucleation sites for iron oxide minerals in all treatments except control (W). MO and MI treatments resulted in well-preserved cell envelopes, while in MIC treatment, cell-shaped holes (bacteriomorphs) prevale. Differences in cell preservation between treatments may result from varying levels of exposure to aqueous iron through cellular permineralization (Southam and Donald, 1999; Levett et al., 2020a).

These observations were accompanied by the development of biofilms on the surface and between grains, resulting in the formation of meniscus-shaped biocements. Biofilms, as highlighted in investigations of natural crusts, play a crucial role in enhancing permeability and water transport, thereby facilitating biogeochemical iron cycling (Benard et al., 2019; Gagen et al., 2019a; Levett et al., 2020a; Paz et al., 2021). In our biocemented blocks from the MO, MI, and MIC treatments, the presence of biofilms embedded in iron oxide indicated successful microbial colonization of the substrate. This colonization likely occurred due to a combination of factors, including the high surface porosity and availability of nutrients. Furthermore, biofilms contribute to iron oxyhydroxide precipitation through local changes in ionic mobility and nucleation of minerals within EPS (Levett et al., 2020a; Paz et al., 2021; Wu et al., 2023).

### 4.3 Microorganism communities

In comparing the microbial composition of iron-reducing cultures with post experiment substrate communities, notable shifts in relative abundance were observed. Specifically, the families Burkholderiaceae and Enterobacteriaceae exhibited decreased abundance in substrates receiving microbial cultures, with only Burkholderiaceae persisting in the control (W) and culture medium (MO) conditions, while Rhizobiaceae increased in all treatments except the control (W). This suggests competitive interactions between the introduced microbial cultures and the native substrate communities.

The influence of carbon sources on microbial communities for accelerating the biogeochemical iron cycle in *canga* systems was investigated. The carbon source plays a crucial role in shaping microbial communities and functional groups. Compared to treatment with iron citrate (III) and acetate (MIC), glucose-enriched treatments (MO and MI) resulted in a higher abundance of *Serratia* spp. from the Enterobacteriaceae family. Notably, some strains of *Serratia* isolated from iron-rich environments demonstrate the ability to couple different carbon source oxidations to iron reduction (Castillo-Zacarías et al., 2020), distinguishing them from other more common strains that rely on iron reduction during glucose and lactate fermentation (Lentini et al., 2012). In this study, it was noted that the increased dissolution of iron oxides in the presence of glucose reflects a pattern in the fermentative ability of *Serratia* spp., consistent with another study indicating a preference of this strain for glucose over acetate when faced with insoluble iron oxides (Castillo-Zacarías et al., 2020).

Additional microbial groups may further have indirectly contributed to the solubilization of iron oxides during the decomposition of organic compounds. Within the glucose treatments (MO and MI), fermenters from the Bacillaceae and Burkholderiaceae groups are highlighted (Valverde et al., 2006; Parker et al., 2018; da Silva et al., 2022). Conversely, in the treatment with Fe(III) citrate/acetate, the Sporolactobacillaceae family is prominent, known for its capacity to produce lactic acid (Chang and Stackebrandt, 2014). This emphasizes that microbial behavior is influenced by the available type of carbon source.

The biocementation of iron-rich fragments involves other bacterial taxa and functional groups. Specifically, this study identified certain groups, such as *Acidovorax* and *Enterobacter*, that have already been recorded as capable of nitrate-reducing activity coupled with Fe(II) oxidation (Carlson et al., 2013; Li et al., 2022). Furthermore, aerobic microorganisms such as Xanthomonadaceae may obtain energy from the reduction of oxygen, coupled with the oxidation of Fe(II) (Neubauer et al., 2002; Grettenberger et al., 2017). The predominant reaction observed in this context was the reduction of nitrates, particularly evident in substrates containing glucose (MO and MI).

These findings highlight the complexity of microbial interactions and metabolic pathways involved in biocementation processes. Understanding the optimization of the biogeochemical iron cycle and its application on larger scales, such as in post mining landscapes, requires further research. Identifying oxidation/reduction cycles is crucial for understanding biocementation processes and evaluating sustainable carbon sources for field applications. Advancements in these areas will enhance our understanding of the mechanisms involved, facilitating the replication and acceleration of the cementing process.

## 5 Conclusion

Our study demonstrated that accelerating the biogeochemical iron cycle is a promising strategy for restoring ferruginous crusts (*canga*). In summary, microorganism feeding and the creation of repeated cycles of anaerobic and aerobic conditions (to stimulate iron reduction and precipitation) are the most important factors triggering the formation of biocemented blocks, accompanied by high rates of textural and mineralogical changes. Although further research and development are required to scale up biocementation in field applications, our study underscores its potential in consolidating iron-rich substrates, highlighting its significance for the restoration of *canga* ecosystems in postmining scenarios.

## Data availability statement

The original contributions presented in the study are included in the article/Supplementary material, further inquiries can be directed to the corresponding author.

## Ethics statement

The manuscript presents research on animals that do not require ethical approval for their study.

## Author contributions

RS: Conceptualization, Data curation, Formal analysis, Investigation, Writing – original draft, Writing – review & editing. AC: Data curation, Formal analysis, Investigation, Writing – review & editing. RA: Writing – review & editing. JB: Conceptualization, Formal analysis, Writing – review & editing. JM: Formal analysis, Writing – review & editing. AL: Writing – review & editing. IP: Data curation, Writing – review & editing, Formal analysis. DC: Data curation, Writing – review & editing. MG: Writing – review & editing, Conceptualization, Data curation, Formal analysis, Funding acquisition, Investigation, Project administration, Validation.
