# Supplementary material for: Enhancing iron biogeochemical cycling for canga ecosystem restoration: insights from microbial stimuli
Source: Front Microbiol. 2024 May 17;15:1352792. doi: 10.3389/fmicb.2024.1352792 (PMC11140077; doi:10.3389/fmicb.2024.1352792)
Supplement: Supplementary file 1 [file Presentation_1.pdf]

Rayara Silva,Aline Figueiredo Cardoso,Rômulo Simões Angélica,José Augusto Bitencourt,Júlio Cezar Fornazier Moreira, Adriano Reis Lucheta,Isabelle Gonçalves de Oliveira Prado, Dalber Ruben Sanchez Candela & Markus Gastauer

Enhancing Iron Biogeochemical Cycling for Canga Ecosystem Restoration: Insights from microbial stimuli

Supplementary material

Frontiers in Microbiology, Microbiological Chemistry and Geomicrobiology,
DOI: 10.3389/fmicb.2024.1352792

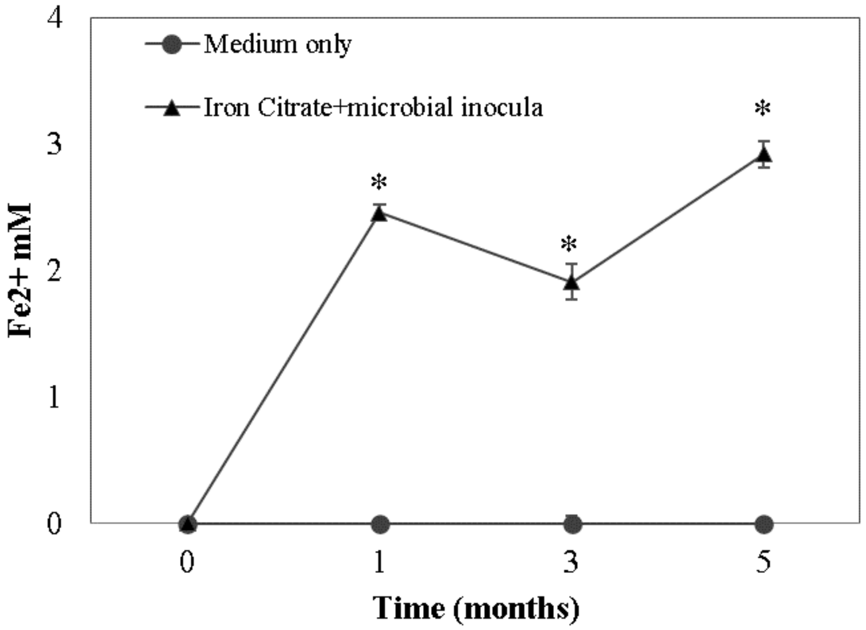

Figure S1. Dissolved Fe(II) concentration in the microbial cultures during the irrigation period of our experiment.

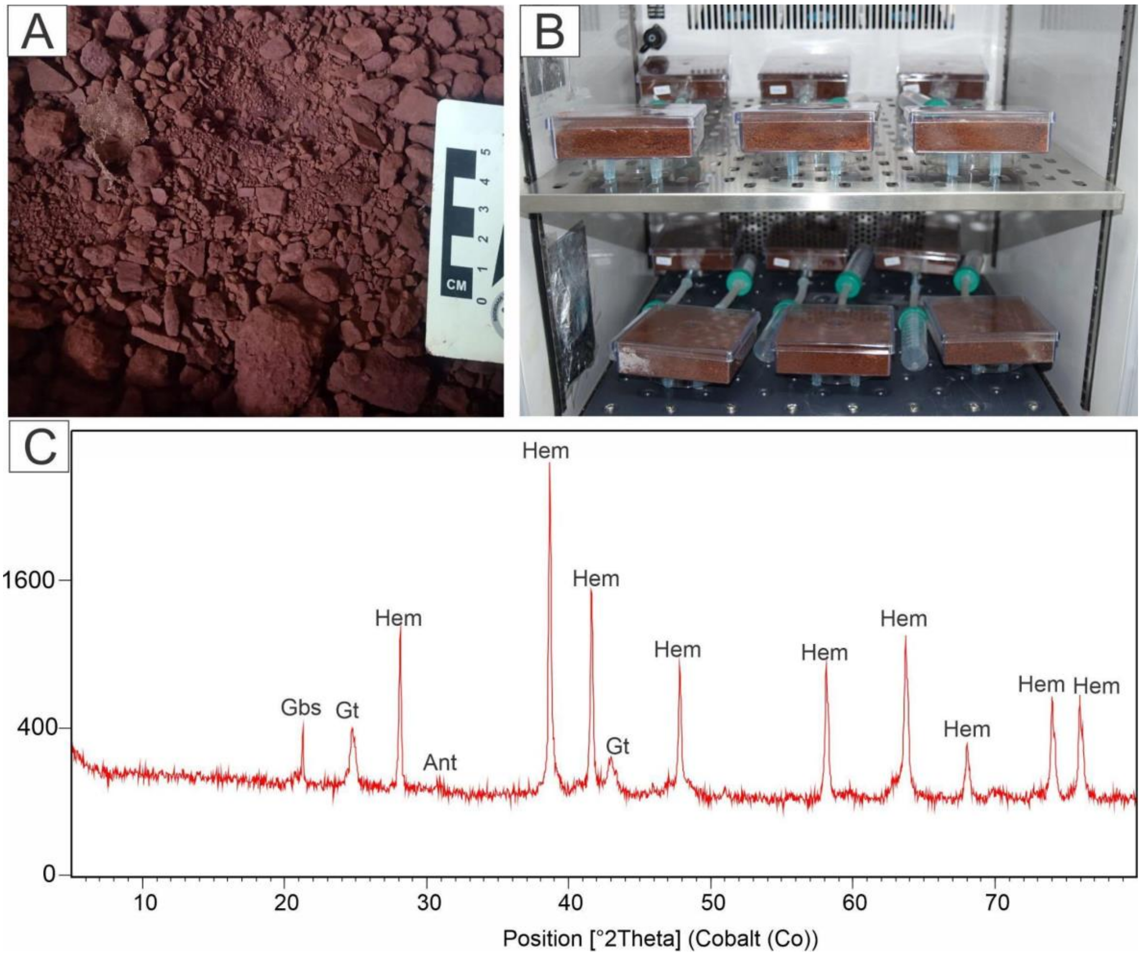

Figure S2. Substrate and overview about the experiment carried out in this study. (A) General aspect of the original substrate, containing crushed ferruginous crust before sieving. (B) Incubator containing acrylic boxes with the sieved substrates, connected to Falcon tubes for drainage of the irrigation solution. (C) X-ray diffractogram of the substrate before treatment. Abbreviations: Gbs (gibbsite); Gt (goethite); Hem (hematite); Ant (anatase).

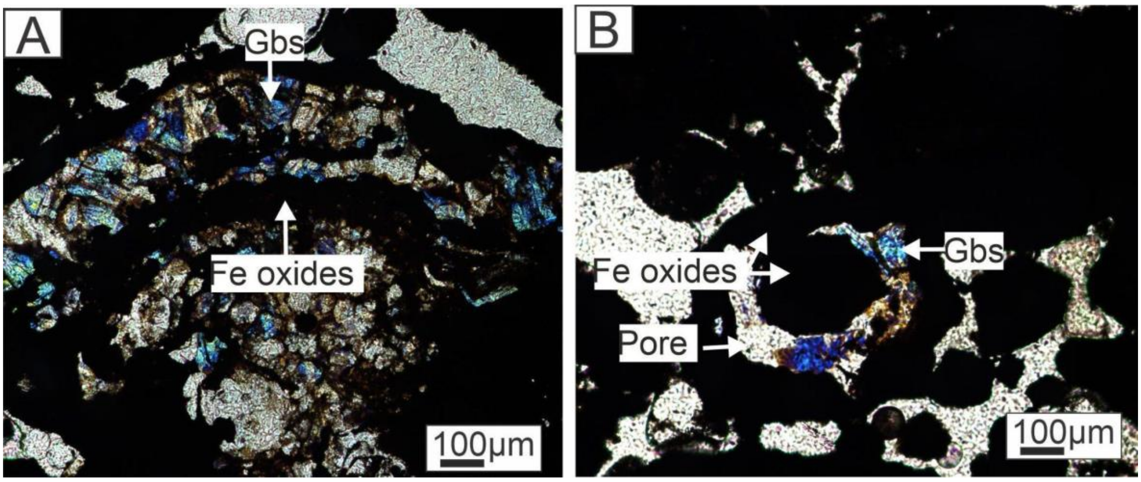

Figure S3. Petrographic images of consolidated samples after treatment with microbial stimuli (MO and MI), Gbs is Gibbsite.

Table S1. Hyperfine parameters obtained from the fit of the 4K Mössbauer spectra: isomer shift (IS), quadrupole splitting (ΔE_Q), linewidth (Γ), magnetic hyperfine field (B_{hf}) and absorption area (A).

Treatments	Site	IS (mm/s)	ΔE_Q (mm/s)	Γ (mm/s)	B_{hf} (T)	A(%)
Water	Hematite	0.494	0.395	0.324	54.27	59.5
	Hematite	0.488	-0.159	0.337	53.57	15.0
	Goethite	0.496	-0.231	0.507	50.12	25.5
Medium Only	Hematite	0.492	0.393	0.297	54.34	64.4
	Hematite	0.498	-0.146	0.337	53.69	15.4
	Goethite	0.494	-0.232	0.433	50.24	20.2
Glucose + Microbial consortium	Hematite	0.494	0.389	0.307	54.34	61.6
	Hematite	0.494	-0.147	0.344	53.71	16.3
	Goethite	0.485	-0.236	0.434	50.16	22.1
Fe (III) Citrate+Microbial consortium	Hematite	0.494	0.396	0.302	54.29	59.3
	Hematite	0.486	-0.134	0.344	53.61	15.2
	Goethite	0.490	-0.233	0.459	50.14	25.5

Table S2. Concentrations (average and standard deviation, n=3) of the major elements (represented as oxides) and trace elements in representative samples from each treatment. LOI (loss on ignition).

Element	Water	Medium Only	Glucose + Microbial consortium	Fe (III) Citrate+Microbial consortium
(wt.%)				
SiO_2	0.700 (±0.36)	0.507 (±0.08)	0.457 (±0.03)	0.450 (±0.03)
TiO_2	0.413 (±0.01)	0.397(±0.01)	0.417 (±0.01)	0.417 (±0.02)
Al_2O_3	3.117 (± 0.02)	2.953 (±0.07)	2.947 (±0.10)	3.023 (±0.05)
Fe_2O_3	90.433(±0.67)	90.333 (±0.25)	90.500 (±0.82)	90.033 (±0.55)
MnO	0.100 (±0.01)	0.090 (± 0.01)	0.090 (±0.00)	0.090 (±0.00)
CaO	0.047 (±0.03)	0.033 (± 0.01)	0.030 (±0.00)	0.027 (±0.01)
Na_2O	<0.100 (±0.00)	0.207 (± 0.03)	0.180 (±0.00)	0.367 (±0.20)
K_2O	0.017 (±0.01)	0.020 (±0.00)	0.020 (±0.00)	0.023 (±0.01)
P_2O_5	0.367 (±0.01)	0.390 (±0.01)	0.407 (±0.02)	0.393 (±0.01)
LOI	3.977 (±0.12)	4.097 (±0.04)	4.160 (±0.20)	4.333 (±0.30)
C	0.193 (±0.01)	0.223 (±0.02)	0.187 (±0.01)	0.243 (±0.04)
S	0.030 (±0.00)	0.050 (±0.01)	0.030 (±0.01)	0.037 (±0.01)
(ppm)				
Be	0.333 (±0.06)	0.267 (±0.06)	0.300 (±0.00)	0.300 (±0.00)
Sc	4.467(±0.06)	3.933 (±0.21)	3.933 (±0.15)	3.833 (±0.06)
V	151.333 (±4.62)	149.333 (±3.51)	152.333 (±13.05)	158.333 (±3.51)
Cr	29.000 (±1.00)	28.333 (±1.53)	30.000 (±1.00)	32.667 (±2.89)
Co	0.967 (±0.12)	3.400 (±1.40)	2.833 (±0.38)	3.700 (±0.36)
Ni	19.000 (±5.29)	22.333 (±12.22)	23.333 (±12.01)	19.667 (±11.59)
Cu	43.400 (±0.53)	47.433 (±1.56)	46.833 (±1.91)	48.200 (±1.42)
Zn	24.667 (±0.58)	45.333 (±13.58)	40.667 (±1.53)	47.667 (±3.06)
Ga	8.767 (±0.25)	8.000 (±0.26)	8.100 (±0.52)	8.333 (±0.12)
Ge	0.800 (±0.10)	0.800 (±0.00)	0.933 (±0.06)	0.700 (±0.00)
As	3.333 (±0.58)	4.000 (±0.00)	3.333 (±0.58)	4.000 (±0.00)
Rb	0.567 (±0.12)	0.533 (±0.15)	0.467 (±0.06)	0.433 (±0.06)
Y	1.940 (±0.24)	1.957 (±0.27)	1.893 (±0.26)	1.827 (±0.09)
Zr	14.300 (±1.49)	14.900 (±0.70)	13.933 (± 1.39)	15.567 (±0.97)
Nb	1.370 (±0.18)	1.353 (±0.18)	1.317 (±0.11)	1.517 (±0.07)
Mo	2.540 (±0.13)	7.363 (±0.55)	6.583 (±0.66)	7.853 (±1.72)
Ag	0.087 (±0.02)	0.073 (±0.02)	0.070 (±0.01)	0.077 (±0.01)
Cd	0.023 (±0.01)	0.023 (±0.01)	0.023 (±0.01)	0.023 (±0.01)
In	0.060 (±0.00)	0.057 (±0.01)	0.053 (±0.01)	0.060 (±0.00)
Sb	0.377 (±0.02)	0.360 (±0.02)	0.357 (±0.02)	0.360 (±0.01)
Cs	0.127 (±0.02)	0.127 (±0.02)	0.123 (±0.02)	0.123 (±0.01)
Ba	19.333 (±3.31)	15.667 (±2.31)	15.667 (±2.31)	15.000 (±1.73)
Hf	0.520 (±0.05)	0.490 (±0.04)	0.487 (±0.05)	0.560 (±0.05)
W	1.067 (±0.47)	2.067 (±0.46)	2.867 (±0.57)	2.800 (±0.17)
Hg	0.100 (±0.02)	0.093 (±0.01)	0.100 (±0.02)	0.097 (±0.01)
Pb	15.500 (±0.72)	14.933 (±1.27)	14.867 (±1.27)	14.900 (±0.26)
Bi	0.197 (±0.01)	0.207 (±0.02)	0.193 (±0.01)	0.200 (±0.00)
Th	2.767 (±0.12)	2.667 (±0.12)	2.633 (±0.06)	2.733 (±0.12)
U	1.113 (±0.02)	1.117 (±0.03)	1.133 (±0.03)	1.153 (±0.08)
La	15.633 (±0.81)	14.333 (±0.45)	14.900 (±0.87)	16.333 (±3.33)
Ce	26.667 (±0.64)	25.933 (±0.06)	25.800 (±0.95)	26.600 (±2.78)
Pr	2.873 (±0.14)	2.627 (±0.18)	2.803 (±0.21)	2.773 (±0.35)
Nd	9.033 (±0.47)	8.200 (±0.44)	8.600 (±1.13)	9.567 (±0.85)
Sm	1.500 (±0.10)	1.200 (±0.30)	1.600 (±0.17)	1.533 (±0.15)
Eu	0.477 (±0.09)	0.410 (±0.08)	0.430 (±0.03)	0.430 (±0.03)
Gd	1.023 (±0.11)	1.090 (±0.09)	1.110 (±0.15)	1.240 (±0.30)
Tb	0.193 (±0.02)	0.157 (±0.03)	0.187 (±0.01)	0.190 (±0.05)
Dy	0.637 (±0.23)	0.577 (±0.11)	0.757 (±0.15)	0.843 (±0.17)
Ho	0.180 (±0.03)	0.173 (±0.02)	0.173 (±0.02)	0.213(±0.04)
Er	0.620 (±0.13)	0.547 (±0.08)	0.673 (±0.06)	0.747(±0.20)
Tm	0.093 (±0.01)	0.100 (±0.03)	0.090 (±0.02)	0.117(±0.03)
Yb	0.500 (±0.01)	0.567 (±0.06)	0.600 (±0.10)	0.567(±0.06)
Lu	0.093 (±0.02)	0.083 (±0.04)	0.103 (±0.01)	0.117(±0.02)

Table S3. Raw chemical data from samples representative of each treatment: Water (W, as a control), Culture medium only (MO), medium+microbial consortium (MI), and medium+microbial consortium +soluble iron (MIC).

			**White**	**W**	**W**	**W**	**MO**	**MO**	**MO**	**MI**	**MI**	**MI**	**MIC**	**MIC**	**MIC**
CSA17V	C	%	0,06	0,19	0,2	0,19	0,22	0,24	0,21	0,18	0,2	0,18	0,23	0,29	0,21
CSA17V	S	%	<0,01	0,03	0,03	0,03	0,06	0,04	0,05	0,03	0,04	0,02	0,04	0,04	0,03
CSA17V	SO_3	%	<0,025	0,069	0,08	0,082	0,147	0,109	0,114	0,071	0,103	0,041	0,093	0,11	0,079
CVA02B	Hg	ppm	<0,05	0,08	0,09	0,07	0,09	0,07	0,07	0,08	0,08	0,07	0,08	0,08	0,07
ICM14B	Ag	ppm	0,03	0,08	0,11	0,07	0,07	0,09	0,06	0,08	0,07	0,06	0,08	0,08	0,07
ICM14B	Al	%	<0,01	1,32	1,35	1,32	1,31	1,33	1,24	1,27	1,16	1,27	1,13	1,35	1,34
ICM14B	As	ppm	<1	3	4	3	4	4	4	3	3	4	4	4	4
ICM14B	B	ppm	<10	<10	<10	<10	<10	<10	<10	<10	<10	<10	<10	<10	<10
ICM14B	Ba	ppm	<5	23	17	18	17	13	17	17	13	17	16	13	16
ICM14B	Be	ppm	<0,1	0,4	0,3	0,3	0,3	0,2	0,3	0,3	0,3	0,3	0,3	0,3	0,3
ICM14B	Bi	ppm	0,03	0,2	0,2	0,19	0,21	0,19	0,22	0,2	0,19	0,19	0,2	0,2	0,2
ICM14B	Ca	%	<0,01	<0,01	<0,01	<0,01	<0,01	<0,01	<0,01	<0,01	<0,01	<0,01	<0,01	<0,01	<0,01
ICM14B	Cd	ppm	0,01	0,03	0,02	0,02	0,03	0,02	0,02	0,02	0,02	0,03	0,03	0,02	0,02
ICM14B	Ce	ppm	0,43	5,62	3,96	4,1	5,53	3,84	4,46	4,84	3,32	3,9	3,5	3,96	3,76
ICM14B	Co	ppm	0,3	1,1	0,9	0,9	5	2,4	2,8	3	3,1	2,4	3,6	4,1	3,4
ICM14B	Cr	ppm	5	28	29	30	28	30	27	29	30	31	36	31	31
ICM14B	Cs	ppm	0,09	0,15	0,11	0,12	0,15	0,12	0,11	0,14	0,1	0,13	0,12	0,12	0,13
ICM14B	Cu	ppm	1,6	43	44	43,2	47,2	46	49,1	47,4	44,7	48,4	47,7	49,8	47,1
ICM14B	Fe	%	0,44	>15	>15	>15	>15	>15	>15	>15	>15	>15	>15	>15	>15
ICM14B	Ga	ppm	<0,1	8,5	9	8,8	7,8	8,3	7,9	7,8	7,8	8,7	8,2	8,4	8,4
ICM14B	Ge	ppm	<0,1	0,9	0,7	0,8	0,8	0,8	0,8	1	0,9	0,9	0,7	0,7	0,7
ICM14B	Hf	ppm	0,06	0,46	0,56	0,54	0,45	0,52	0,5	0,43	0,51	0,52	0,51	0,6	0,57
ICM14B	Hg	ppm	<0,01	0,09	0,12	0,09	0,1	0,1	0,08	0,1	0,12	0,08	0,09	0,11	0,09
ICM14B	In	ppm	<0,02	0,06	0,06	0,06	0,06	0,06	0,05	0,05	0,05	0,06	0,06	0,06	0,06
ICM14B	K	%	<0,01	<0,01	<0,01	<0,01	0,01	0,01	0,01	0,01	0,01	0,01	0,01	0,01	<0,01
ICM14B	La	ppm	0,2	2,9	2	2	2,9	2	2,1	2,5	1,7	2	1,8	2	2
ICM14B	Li	ppm	<1	<1	<1	<1	<1	<1	<1	<1	<1	<1	<1	<1	<1
ICM14B	Lu	ppm	0,02	0,02	0,02	0,02	0,03	0,02	0,02	0,02	0,02	0,02	0,02	0,02	0,02
ICM14B	Mg	%	<0,01	<0,01	<0,01	<0,01	<0,01	<0,01	<0,01	<0,01	<0,01	<0,01	<0,01	<0,01	<0,01
ICM14B	Mn	ppm	78	448	417	461	414	385	564	448	356	407	487	380	419
ICM14B	Mo	ppm	0,21	2,4	2,65	2,57	7,64	6,73	7,72	6,98	6,95	5,82	7,23	9,8	6,53
ICM14B	Na	%	0,02	<0,01	<0,01	<0,01	0,19	0,14	0,14	0,15	0,15	0,09	0,21	0,44	0,22
ICM14B	Nb	ppm	0,12	1,2	1,56	1,35	1,17	1,53	1,36	1,2	1,33	1,42	1,47	1,6	1,48
ICM14B	Ni	ppm	6,6	<0,5	<0,5	<0,5	<0,5	<0,5	<0,5	<0,5	<0,5	<0,5	<0,5	<0,5	<0,5
ICM14B	P	ppm	<50	1307	1379	1317	1484	1459	1404	1417	1482	1641	1535	1554	1526
ICM14B	Pb	ppm	0,3	16,3	14,9	15,3	16,3	14,7	13,8	15,7	13,4	15,5	15	15,1	14,6
ICM14B	Rb	ppm	0,6	0,7	0,5	0,5	0,7	0,5	0,4	0,5	0,4	0,5	0,5	0,4	0,4
ICM14B	Re	ppm	<0,1	<0,1	<0,1	<0,1	<0,1	<0,1	<0,1	<0,1	<0,1	<0,1	<0,1	<0,1	<0,1
ICM14B	S	%	<0,01	0,02	0,02	0,02	0,06	0,03	0,03	0,02	0,02	<0,01	0,02	0,04	0,02
ICM14B	Sb	ppm	0,16	0,4	0,37	0,36	0,38	0,35	0,35	0,36	0,34	0,37	0,36	0,35	0,37
ICM14B	Sc	ppm	<0,1	4,5	4,5	4,4	4	4,1	3,7	3,9	3,8	4,1	3,8	3,9	3,8
ICM14B	Se	ppm	<1	<1	<1	<1	<1	<1	<1	<1	<1	<1	<1	1	<1
ICM14B	Sn	ppm	<0,3	1	1	1,1	1	1	1	1	1	1,1	1,1	1,1	1,1
ICM14B	Sr	ppm	4,2	<0,5	<0,5	<0,5	<0,5	<0,5	<0,5	<0,5	<0,5	<0,5	<0,5	<0,5	<0,5
ICM14B	Ta	ppm	<0,05	<0,05	<0,05	<0,05	<0,05	<0,05	<0,05	<0,05	<0,05	<0,05	<0,05	<0,05	<0,05
ICM14B	Tb	ppm	<0,02	0,05	0,03	0,04	0,05	0,03	0,04	0,04	0,03	0,04	0,03	0,04	0,04
ICM14B	Te	ppm	<0,05	<0,05	<0,05	<0,05	<0,05	<0,05	<0,05	<0,05	<0,05	<0,05	<0,05	<0,05	<0,05
ICM14B	Th	ppm	0,1	2,9	2,7	2,7	2,8	2,6	2,6	2,6	2,7	2,6	2,6	2,8	2,8
ICM14B	Ti	%	<0,01	0,07	0,07	0,07	0,06	0,07	0,07	0,06	0,06	0,07	0,07	0,07	0,07

ICM14B	Tl	ppm	0,03	<0,02	<0,02	<0,02	<0,02	<0,02	<0,02	<0,02	<0,02	<0,02	<0,02	<0,02
ICM14B	U	ppm	<0,05	1,13	1,12	1,09	1,11	1,09	1,15	1,15	1,1	1,15	1,07	1,23	1,16
ICM14B	V	ppm	<1	146	154	154	146	153	149	148	142	167	155	162	158
ICM14B	W	ppm	0,1	1,6	0,9	0,7	2,6	1,8	1,8	2,7	2,4	3,5	2,9	2,9	2,6
ICM14B	Y	ppm	0,07	2,22	1,79	1,81	2,26	1,77	1,84	2,16	1,65	1,87	1,73	1,89	1,86
ICM14B	Yb	ppm	<0,1	0,1	0,1	0,1	0,2	0,1	0,1	0,1	0,1	0,1	0,1	0,1	0,1
ICM14B	Zn	ppm	<1	25	24	25	61	38	37	41	39	42	47	51	45
ICM14B	Zr	ppm	<0,5	12,6	15,4	14,9	14,2	15,6	14,9	12,4	14,3	15,1	14,5	16,4	15,8
IMS95A	Ce	ppm	0,1	26,2	27,4	26,4	25,9	25,9	26	24,9	25,7	26,8	29,8	24,8	25,2
IMS95A	Co	ppm	<0,5	1,4	1,5	1,6	4,7	2,4	2,7	3	3,7	2,7	4,3	4,4	3,3
IMS95A	Cs	ppm	<0,05	<0,05	<0,05	<0,05	<0,05	<0,05	<0,05	<0,05	<0,05	<0,05	<0,05	<0,05	<0,05
IMS95A	Cu	ppm	<5	60	61	62	67	62	68	67	73	64	71	66	66
IMS95A	Dy	ppm	<0,05	0,89	0,57	0,45	0,68	0,46	0,59	0,59	0,8	0,88	1,03	0,7	0,8
IMS95A	Er	ppm	0,16	0,77	0,55	0,54	0,61	0,46	0,57	0,69	0,72	0,61	0,98	0,6	0,66
IMS95A	Eu	ppm	<0,05	0,5	0,55	0,38	0,48	0,32	0,43	0,43	0,4	0,46	0,41	0,42	0,46
IMS95A	Ga	ppm	0,8	8,6	9,8	9,4	9,3	9,1	8,5	8,4	9	10,2	11,1	9,5	9,8
IMS95A	Gd	ppm	<0,05	0,91	1,12	1,04	1,01	1,18	1,08	0,96	1,11	1,26	1,48	1,34	0,9
IMS95A	Hf	ppm	0,73	2,83	1,93	2,26	2,28	2,19	2	2,14	2,4	2,02	2,14	1,81	2,38
IMS95A	Ho	ppm	0,05	0,18	0,21	0,15	0,2	0,16	0,16	0,16	0,2	0,16	0,26	0,19	0,19
IMS95A	La	ppm	<0,1	16,1	16,1	14,7	14,8	13,9	14,3	13,9	15,4	15,4	20,1	13,8	15,1
IMS95A	Lu	ppm	<0,05	0,09	0,08	0,11	0,07	0,05	0,13	0,1	0,11	0,1	0,13	0,12	0,1
IMS95A	Mo	ppm	<2	6	5	4	10	8	10	9	11	6	13	11	9
IMS95A	Nb	ppm	4,41	5,85	4,62	5,05	5,17	4,23	4,3	4,45	4,83	4,55	5,59	3,91	3,82
IMS95A	Nd	ppm	0,2	8,5	9,4	9,2	8,5	7,7	8,4	7,9	8	9,9	10,4	8,7	9,6
IMS95A	Ni	ppm	9	21	23	13	25	9	33	35	24	11	33	12	14
IMS95A	Pr	ppm	0,07	2,89	3	2,73	2,7	2,42	2,76	2,61	2,78	3,02	3,14	2,45	2,73
IMS95A	Rb	ppm	0,7	2	1,5	1,2	1,6	1,9	1,8	1,3	1,4	1,4	2,5	1,7	2,2
IMS95A	Sm	ppm	<0,1	1,5	1,4	1,6	1,2	0,9	1,5	1,5	1,5	1,8	1,7	1,5	1,4
IMS95A	Sn	ppm	<0,3	<0,3	<0,3	<0,3	<0,3	<0,3	<0,3	<0,3	<0,3	2,3	0,4	0,5	2,2
IMS95A	Ta	ppm	1,75	2,15	1,73	1,29	1,01	0,97	0,84	0,75	0,66	0,61	0,66	0,6	0,59
IMS95A	Tb	ppm	<0,05	0,2	0,21	0,17	0,18	0,12	0,17	0,2	0,18	0,18	0,23	0,2	0,14
IMS95A	Th	ppm	0,3	5,8	5,4	5,1	5,1	4,8	4,8	5	5,1	4,6	5,4	4,7	4,7
IMS95A	Tl	ppm	<0,5	<0,5	<0,5	<0,5	<0,5	<0,5	<0,5	<0,5	<0,5	<0,5	<0,5	<0,5	<0,5
IMS95A	Tm	ppm	<0,05	0,09	0,09	0,1	0,13	0,07	0,1	0,1	0,1	0,07	0,15	0,11	0,09
IMS95A	U	ppm	0,08	1,48	1,51	1,61	1,53	1,48	1,51	1,64	1,55	1,46	1,7	1,53	1,61
IMS95A	W	ppm	<0,1	4,8	4,4	3,6	4,2	2,9	3,9	4,6	6,9	6,3	5,7	4,8	4,1
IMS95A	Y	ppm	1,55	7,33	7,6	7,16	7,04	6,47	6,95	6,91	7,9	7,47	9,98	7,19	7,43
IMS95A	Yb	ppm	0,1	0,5	0,5	0,5	0,6	0,6	0,5	0,6	0,7	0,5	0,6	0,5	0,6
ISE03A	F	ppm	41	<30	34	<30	<30	<30	<30	<30	33	<30	<30	<30	39
XRF79C	SiO_2	%	99	1,12	0,51	0,47	0,53	0,42	0,57	0,48	0,43	0,46	0,47	0,47	0,41
XRF79C	Al_2O_3	%	0,11	3,14	3,11	3,1	2,91	3,03	2,92	2,83	2,98	3,03	2,99	3	3,08
XRF79C	Fe_2O_3	%	0,66	90	90,1	91,2	90,6	90,1	90,3	90,7	91,2	89,6	90,4	89,4	90,3
XRF79C	CaO	%	0,02	0,08	0,03	0,03	0,03	0,03	0,04	0,03	0,03	0,03	0,03	0,02	0,03
XRF79C	MgO	%	<0,1	<0,1	<0,1	<0,1	<0,1	<0,1	<0,1	<0,1	<0,1	<0,1	<0,1	<0,1	<0,1
XRF79C	TiO_2	%	0,01	0,41	0,41	0,42	0,41	0,39	0,39	0,41	0,42	0,42	0,44	0,4	0,41
XRF79C	P_2O_5	%	<0,01	0,37	0,37	0,36	0,4	0,38	0,39	0,39	0,41	0,42	0,4	0,39	0,39
XRF79C	Na_2O	%	<0,1	<0,1	<0,1	<0,1	0,24	0,2	0,18	0,18	0,18	<0,1	0,25	0,6	0,25
XRF79C	K_2O	%	<0,01	0,03	0,01	0,01	0,02	0,02	0,02	0,02	0,02	0,02	0,02	0,03	0,02
XRF79C	MnO	%	<0,01	0,09	0,1	0,11	0,09	0,08	0,1	0,09	0,09	0,09	0,09	0,09	0,09
XRF79C	BaO	%	<0,01	<0,01	<0,01	<0,01	<0,01	<0,01	<0,01	<0,01	<0,01	<0,01	<0,01	<0,01	<0,01
XRF79C	Cr_2O_3	%	<0,01	<0,01	<0,01	<0,01	<0,01	<0,01	<0,01	<0,01	<0,01	<0,01	<0,01	<0,01	<0,01
XRF79C	SrO	%	0,02	<0,01	<0,01	<0,01	<0,01	<0,01	<0,01	<0,01	<0,01	<0,01	<0,01	<0,01	<0,01
PHY01E	LOI	%	0,08	3,84	4,05	4,04	4,1	4,06	4,13	3,94	4,32	4,22	4,08	4,67	4,25

Table S4. Taxonomic annotations and mean number of reads (n=3) of the OTUs identified by Illumina 16S rRNA sequencing extracted from substrates and microbial cultures; only bacterial data was accessed. Treatments: Water (W, as a control), Culture medium only (MO), medium+microbial consortium (MI), and medium+microbial consortium +soluble iron (MIC).

OTUId	Kingdom	Phylum	Class	Order	Family	Genus	Microbial cultures		Substrate			
							MI	MIC	W	MO	MI	MIC
OTU755874832	Bacteria	Proteobacteria	Gammaproteobacteria	Enterobacterial	Enterobacteriaceae	Serratia	12073	23464.67	47	267.67	1098.67	137.33
OTU199185381	Bacteria	Proteobacteria	Alphaproteobacteria	Rhizobiales	Rhizobiaceae	-	2974	5894.33	6.33	6656	8450	3448.67
OTU757476480	Bacteria	Proteobacteria	Gammaproteobacteria	Betaproteobacterial	Burkholderiaceae	Achromobacter	2201.33	8787	22.67	3075	738	4.67
OTU844384522	Bacteria	Proteobacteria	Gammaproteobacteria	Enterobacterial	Enterobacteriaceae	-	348	12053.33	28	122.67	102.33	22.33
OTU774817072	Bacteria	Proteobacteria	Alphaproteobacteria	Rhizobiales	Rhizobiaceae	Ensifer	1470	2828.33	3.33	2681	3900.33	1709
OTU514384367	Bacteria	Proteobacteria	Alphaproteobacteria	Rhizobiales	Rhizobiaceae	-	1296.33	2536.33	2.33	2828	3532	1325.67
OTU955568123	Bacteria	Proteobacteria	Gammaproteobacteria	Enterobacterial	Enterobacteriaceae	Serratia	3605.33	7028.33	10	59.67	270.67	32.33
OTU613423798	Bacteria	Actinobacteria	Actinobacteria	Micrococcal	Microbacteriaceae	Leucobacter	2.33	10	879.33	2075.33	4477.33	3068
OTU27569162	Bacteria	Proteobacteria	Gammaproteobacteria	Enterobacterial	Enterobacteriaceae	Serratia	2418.67	7304.67	2.67	64.33	64.33	20
OTU444295085	Bacteria	Proteobacteria	Alphaproteobacteria	Rhizobiales	Rhizobiaceae	Ensifer	1292.67	2591.67	3.67	2015.67	2768.67	1174.67

OTU281024715	Bacteria	Proteobacteria	Gammaproteobacteria	Enterobacterial	Enterobacteriaceae	Serratia	3128.33	5975	9.67	59.67	201	14.67
OTU474272912	Bacteria	Proteobacteria	Gammaproteobacteria	Enterobacterial	Enterobacteriaceae	Serratia	3093.67	5885	8.67	62.33	262.67	39.67
OTU740532606	Bacteria	Proteobacteria	Gammaproteobacteria	Enterobacterial	Enterobacteriaceae	Serratia	2786	5258.33	9	45.33	206.67	25.67
OTU416286231	Bacteria	Proteobacteria	Gammaproteobacteria	Enterobacterial	Enterobacteriaceae	-	205.33	7099.67	15	71	62.67	12.33
OTU27254687	Bacteria	Proteobacteria	Gammaproteobacteria	Enterobacterial	Enterobacteriaceae	Serratia	2510.67	4473.67	5.33	45.33	220	26.33
OTU437079873	Bacteria	Proteobacteria	Alphaproteobacteria	Rhizobiales	Rhizobiaceae	-	1102.67	2147	4.67	1165	1612	724.33
OTU249565946	Bacteria	Proteobacteria	Alphaproteobacteria	Rhizobiales	Rhizobiaceae	-	1062.67	2041.67	1	436.67	1779.33	1153.33
OTU262816563	Bacteria	Proteobacteria	Alphaproteobacteria	Rhizobiales	Beijerinckiaceae	Methylobacterium	0	0	6344	2	0.67	0.33
OTU92274122	Bacteria	Proteobacteria	Gammaproteobacteria	Enterobacterial	Enterobacteriaceae	Enterobacter	145.33	5489.33	14.67	88	73.67	16.33
OTU797657709	Bacteria	Proteobacteria	Gammaproteobacteria	Betaproteobacterial	Burkholderiaceae	Delftia	2936	1622	23.33	497.67	14	1
OTU626242069	Bacteria	Proteobacteria	Gammaproteobacteria	Enterobacterial	Enterobacteriaceae	Enterobacter	112.33	4515.33	9	45.67	43.33	8.67
OTU786806734	Bacteria	Proteobacteria	Alphaproteobacteria	Rhizobiales	Rhizobiaceae	-	810	1653.67	2.33	268	1094	626.33
OTU122399278	Bacteria	Proteobacteria	Gammaproteobacteria	Enterobacterial	Enterobacteriaceae	-	91	3267.67	6.67	26	26.67	5.67
OTU11119769	Bacteria	Proteobacteria	Gammaproteobacteria	Betaproteobacterial	Burkholderiaceae	Achromobacter	508.33	1933	7.33	728	173.33	2.67

OTU525787719	Bacteria	Proteobacteria	Gammaproteobacteria	Betaproteobacterial	Burkholderiaceae	Achromobacter	517.33	2005.33	3.33	548	127.67	1.67
OTU811406262	Bacteria	Actinobacteria	Actinobacteria	Propionibacterial	Nocardioidaceae	Nocardioids	0	0	3168.33	0	0	0
OTU470865673	Bacteria	Proteobacteria	Gammaproteobacteria	Enterobacterial	Enterobacteriaceae	Serratia	995.33	2014.67	4.67	24.67	72.67	11
OTU395026531	Bacteria	Firmicutes	Bacilli	Bacillales	Bacillaceae	Bacillus	0	0	0	0	2744	320
OTU118585768	Bacteria	Proteobacteria	Gammaproteobacteria	Enterobacterial	Enterobacteriaceae	-	1539	977.33	7	148.67	1.67	221.67
OTU238906161	Bacteria	Proteobacteria	Gammaproteobacteria	Enterobacterial	Enterobacteriaceae	Serratia	892.67	1796.67	1.67	16	61.33	9.33
OTU850044365	Bacteria	Proteobacteria	Gammaproteobacteria	Xanthomonadales	Xanthomonadaceae	Stenotrophomonas	0.33	6.67	14.67	2384.67	353.67	0
OTU49694034	Bacteria	Proteobacteria	Gammaproteobacteria	Betaproteobacterial	Burkholderiaceae	Achromobacter	439.33	1694	5.33	446.67	98	0.67
OTU324021995	Bacteria	Proteobacteria	Gammaproteobacteria	Enterobacterial	Enterobacteriaceae	Serratia	851.33	1696.67	4.67	26.67	84	9.67
OTU150441982	Bacteria	Proteobacteria	Gammaproteobacteria	Enterobacterial	Enterobacteriaceae	Serratia	841	1735.67	2	13.67	62.33	12
OTU26463333	Bacteria	Proteobacteria	Gammaproteobacteria	Enterobacterial	Enterobacteriaceae	Serratia	821.33	1730.67	2	14.33	68.67	11.33
OTU630181490	Bacteria	Proteobacteria	Gammaproteobacteria	Betaproteobacterial	Burkholderiaceae	Achromobacter	453.33	1694.33	2.67	406	80.33	0.33
OTU951836145	Bacteria	Proteobacteria	Gammaproteobacteria	Enterobacterial	Enterobacteriaceae	Serratia	787.33	1680.33	1.33	11	43.33	5.67
OTU308607689	Bacteria	Proteobacteria	Gammaproteobacteria	Enterobacterial	Enterobacteriaceae	Serratia	557	1851.67	0.67	25.33	14	11.33

OTU699505644	Bacteria	Proteobacteria	Alphaproteobacteria	Sphingomonadales	Sphingomonadaceae	Sphingomonas	1.67	0	2391.67	8	0	1.33
OTU887331756	Bacteria	Proteobacteria	Gammaproteobacteria	Betaproteobacterial	Burkholderiaceae	Burkholderia-Caballeronia-Paraburkholderia	2	2222	1.67	0	12.33	0
OTU511145617	Bacteria	Proteobacteria	Alphaproteobacteria	Rhizobiales	Beijerinckiaceae	Methylobacterium	0	0	2158.67	0	0	0
OTU32264837	Bacteria	Firmicutes	Bacilli	Bacillales	Sporolactobacillaceae	Alkalicoccus	0	0	0	0	0.33	2105
OTU620307136	Bacteria	Firmicutes	Bacilli	Bacillales	Bacillaceae	Bacillus	0	0	0	0	2049.67	0
OTU912091633	Bacteria	Proteobacteria	Gammaproteobacteria	Enterobacterial	Enterobacteriaceae	Serratia	483	1472	0.33	9.33	7.67	4.33
OTU350995993	Bacteria	Proteobacteria	Gammaproteobacteria	Enterobacterial	Enterobacteriaceae	Serratia	429	1405	0.67	14.33	10.33	6
OTU946226090	Bacteria	Proteobacteria	Gammaproteobacteria	Enterobacterial	Enterobacteriaceae	Serratia	574.33	1197.33	2.67	10.33	31.67	6.33
OTU488591389	Bacteria	Actinobacteria	Actinobacteria	Micrococcal	Microbacteriaceae	-	0	2	169.33	363.67	761.67	523.33
OTU124380547	Bacteria	Proteobacteria	Gammaproteobacteria	Betaproteobacterial	Burkholderiaceae	Burkholderia-Caballeronia-Paraburkholderia	5.67	0	1739.33	1.33	0	0
OTU958873113	Bacteria	Firmicutes	Bacilli	Bacillales	Bacillaceae	Bacillus	0	0	0	1711	25	0.67
OTU536591318	Bacteria	Actinobacteria	Actinobacteria	Micrococcal	Micrococcaceae	-	0	0	1687.33	2.67	0.67	0

OTU911523834	Bacteria	Proteobacteria	Gammaproteobacteria	Betaproteobacterial	Burkholderiaceae	Delftia	819	468.67	3.33	161.67	2.33	0.33
OTU327182815	Bacteria	Proteobacteria	Gammaproteobacteria	Enterobacterial	Enterobacteriaceae	Klebsiella	763	485.67	4.67	70.33	0.33	100
OTU844210746	Bacteria	Firmicutes	Bacilli	Bacillales	Bacillaceae	Bacillus	0	0	0	1256	21.67	0
OTU900962403	Bacteria	Actinobacteria	Actinobacteria	Micrococcal		-	0	0	203.33	1024.33	17.33	0.33
OTU301488314	Bacteria	Proteobacteria	Gammaproteobacteria	Enterobacterial	Enterobacteriaceae	-	623.67	403.67	3.67	72.33	1.33	100
OTU100559206	Bacteria	Proteobacteria	Alphaproteobacteria	Sphingomonadales	Sphingomonadaceae	Sphingomonas	0.67	0.33	1039.6<br>7	3.33	0	1
OTU791226492	Bacteria	Firmicutes	Bacilli	Bacillales	Bacillaceae	Bacillus	0	0	0	0	874.67	111.33
OTU745433186	Bacteria	Firmicutes	Bacilli	Bacillales	Bacillaceae	Bacillus	0	0	0	0	871.67	95.67
OTU591724868	Bacteria	Proteobacteria	Gammaproteobacteria	Betaproteobacterial	Burkholderiaceae	Ralstonia	0	0	967	0	0	0
OTU658894669	Bacteria	Proteobacteria	Alphaproteobacteria	Rhizobiales	Beijerinckiaceae	Methylobacterium	0	0	933.67	0.67	0.33	0
OTU799178417	Bacteria	Proteobacteria	Gammaproteobacteria	Betaproteobacterial	Burkholderiaceae	Comamonas	99.33	815	0	0	2.67	0.33
OTU274226634	Bacteria	Proteobacteria	Alphaproteobacteria	Rhizobiales	Rhizobiaceae	Ensifer	25	32.33	1.67	257	380.67	158.33
OTU827300806	Bacteria	Proteobacteria	Gammaproteobacteria	Betaproteobacterial	Burkholderiaceae	Delftia	500	283.67	0.67	62.67	0.33	0.33
OTU213686141	Bacteria	Proteobacteria	Gammaproteobacteria	Betaproteobacterial	Burkholderiaceae	Achromobacter	57	585.67	0.67	97.33	89.33	1.67

OTU811180894	Bacteria	Actinobacteria	Actinobacteria	Micrococcal	Micrococcaceae	Synomonas	0	0	712.67	0.67	0	0
OTU273014628	Bacteria	Proteobacteria	Gammaproteobacteria	Betaproteobacterial	Burkholderiaceae	Achromobacter	48.67	553.33	0	53.67	55	0
OTU122894787	Bacteria	Proteobacteria	Alphaproteobacteria	Rhizobiales	Rhizobiaceae	-	162.67	410.67	0	39.67	57	34.33
OTU199390458	Bacteria	Actinobacteria	Actinobacteria	Micrococcal	Micrococcaceae	Synomonas	0	0	687.67	0.67	0	0
OTU347429467	Bacteria	Firmicutes	Bacilli	Bacillales	Bacillaceae	Bacillus	0	0	0	0	609	73
OTU957893310	Bacteria	Proteobacteria	Gammaproteobacteria	Betaproteobacterial	Burkholderiaceae	Achromobacter	49.67	385	0.33	73	68.33	0.33
OTU963132214	Bacteria	Proteobacteria	Gammaproteobacteria	Betaproteobacterial	Burkholderiaceae	Achromobacter	37.33	383	1	83.33	66.67	0.67
OTU73970550	Bacteria	Proteobacteria	Gammaproteobacteria	Xanthomonadales	Xanthomonadaceae	Stenotrophomonas	2.33	23	0	539	4.67	0
OTU195221956	Bacteria	Proteobacteria	Alphaproteobacteria	Rhizobiales	Xanthobacteraceae	-	4	0	545.67	0	0	0
OTU609468510	Bacteria	Proteobacteria	Gammaproteobacteria	Enterobacterial	Enterobacteriaceae	Serratia	139.33	395.33	0	0.67	9	2.33
OTU427484060	Bacteria	Proteobacteria	Gammaproteobacteria	Betaproteobacterial	Burkholderiaceae	Delftia	309	176	1.67	54.33	1	0
OTU559502822	Bacteria	Firmicutes	Bacilli	Lactobacillus	Enterococcaceae	Enterococci	28	196.67	0	0	14.33	278
OTU951876665	Bacteria	Proteobacteria	Gammaproteobacteria	Enterobacterial	Enterobacteriaceae	-	281.33	186.67	0.67	18.67	0.33	29
OTU624766999	Bacteria	Proteobacteria	Alphaproteobacteria	Rhizobiales	Beijerinckiaceae	Methylobacterium	0.33	0	514.67	0	0	0
OTU355376262	Bacteria	Proteobacteria	Alphaproteobacteria	Rhizobiales	Rhizobiaceae	Ensifer	17.67	23.67	3.33	137.67	202	101.67

OTU377631900	Bacteria	Proteobacteria	Alphaproteobacteria	Rhizobiales	Rhizobiaceae	-	5.67	8.67	0.33	142.67	207.67	86
OTU903995175	Bacteria	Firmicutes	Bacilli	Bacillales	Bacillaceae	Bacillus	0	0	0	0	388.67	47.67
OTU453793457	Bacteria	Proteobacteria	Alphaproteobacteria	Rhizobiales	Beijerinckiaceae	Methylobacterium	0	0	430.33	0	0	0
OTU666746028	Bacteria	Proteobacteria	Gammaproteobacteria	Enterobacterial	Enterobacteriaceae	Serratia	153.67	256.67	0.67	3.67	13.33	1.33
OTU679350989	Bacteria	Firmicutes	Bacilli	Bacillales	Bacillaceae	Bacillus	0	0	0	0	0	426.33
OTU636561175	Bacteria	Bacteroidetes	Bacteroidia	Chitinophagales	Chitinophagaceae	Asinibacterium	0	0	424.67	0	0	0
OTU464018897	Bacteria	Firmicutes	Bacilli	Bacillales	Bacillaceae	Bacillus	0	0	0	385.67	6.33	0.33
OTU377201673	Bacteria	Actinobacteria	Actinobacteria	Streptomycetales	Streptomycetaceae	Streptomyces	0	0	353.67	13.33	0	0
OTU972347779	Bacteria	Firmicutes	Bacilli	Bacillales	Staphylococcus	Staphylococcus	0	0	0	358	0	0
OTU253898171	Bacteria	Firmicutes	Bacilli	Bacillales	Bacillaceae	Bacillus	0	0	0	0	356.33	0
OTU549991370	Bacteria	Proteobacteria	Gammaproteobacteria	Betaproteobacterial	Burkholderiaceae	Achromobacter	24.33	253.67	0	31	30	0
OTU898192860	Bacteria	Proteobacteria	Gammaproteobacteria	Betaproteobacterial	Burkholderiaceae	Achromobacter	61.33	59.33	1.67	166.33	35	0.67
OTU289165228	Bacteria	Proteobacteria	Gammaproteobacteria	Betaproteobacterial	Burkholderiaceae	Acidovorax	75.33	204	16.67	3	20.33	0
OTU945421907	Bacteria	Proteobacteria	Alphaproteobacteria	Rhizobiales	Rhizobiaceae	-	57	89.33	0.33	51.33	73.67	34.33
OTU707361475	Bacteria	Proteobacteria	Gammaproteobacteria	Xanthomonadales	Xanthomonadaceae	Stenotrophomonas	0	0	2	262	41.33	0

OTU469056780	Bacteria	Proteobacteria	Gammaproteobacteria	Enterobacterial	Enterobacteriaceae	Klebsiella	14.67	283.67	0	0	1	0
OTU988953895	Bacteria	Proteobacteria	Alphaproteobacteria	Rhizobiales	Rhizobiaceae	-	45.33	84.67	0.67	54	73.67	40
OTU214130223	Bacteria	Actinobacteria	Actinobacteria	Micrococcal	Micrococcaceae	Synomonas	0	0	295.67	1	0	0
OTU386987021	Bacteria	Proteobacteria	Gammaproteobacteria	Enterobacterial	Enterobacteriaceae	-	37	256.67	0	0	1.33	0.33
OTU109648143	Bacteria	Proteobacteria	Alphaproteobacteria	Rhizobiales	Rhizobiaceae	-	6.33	3	223.67	37	20	1
OTU11369194	Bacteria	Firmicutes	Bacilli	Bacillales	Sporolactobacillaceae	Alkalicoccus	0	0	0	0	0	286.67
OTU814858199	Bacteria	Actinobacteria	Actinobacteria	Frankiales	Geodermatophilaceae	Antricoccus	1	3.33	255.33	2.67	14.33	5.33
OTU309332861	Bacteria	Proteobacteria	Gammaproteobacteria	Betaproteobacterial	Burkholderiaceae	Delftia	161.33	73	1	38.33	0.33	0
OTU785955268	Bacteria	Proteobacteria	Gammaproteobacteria	Enterobacterial	Enterobacteriaceae	Serratia	90	168	0.33	3.67	9.33	1
OTU976560094	Bacteria	Proteobacteria	Gammaproteobacteria	Enterobacterial	Enterobacteriaceae	Serratia	85.33	174.33	1	2	7.33	0.67
OTU934649190	Bacteria	Proteobacteria	Alphaproteobacteria	Rhizobiales	Rhizobiaceae	Ensifer	32.33	69.67	0	56.33	66.33	32.67
OTU562163905	Bacteria	Proteobacteria	Gammaproteobacteria	Enterobacterial	Enterobacteriaceae	Serratia	74	149.67	1.67	5.67	19	5
OTU311610153	Bacteria	Proteobacteria	Gammaproteobacteria	Enterobacterial	Enterobacteriaceae	Enterobacter	126	84	1.33	15.33	0.33	27.33
OTU681670971	Bacteria	Proteobacteria	Alphaproteobacteria	Rhizobiales	Rhizobiaceae	-	35	67.33	0	43	72.33	31.33
OTU946777962	Bacteria	Proteobacteria	Alphaproteobacteria	Rhizobiales	Rhizobiaceae	-	25.33	51.33	0	50.33	76	36.33

OTU709744372	Bacteria	Proteobacteria	Gammaproteobacteria	Enterobacterial	Enterobacteriaceae	Serratia	142.67	69.67	0.33	4	12.67	2.33
OTU216287096	Bacteria	Proteobacteria	Gammaproteobacteria	Enterobacterial	Enterobacteriaceae	Enterobacter	4	221.67	0	2.33	2	0.33
OTU1364231	Bacteria	Proteobacteria	Gammaproteobacteria	Enterobacterial	Enterobacteriaceae	Serratia	51	98.33	2.33	17	49.67	11.67
OTU12432736	Bacteria	Proteobacteria	Gammaproteobacteria	Enterobacterial	Enterobacteriaceae	-	7.67	216.33	0.33	1.67	1	0.33
OTU279742192	Bacteria	Proteobacteria	Alphaproteobacteria	Rhizobiales	Xanthobacteraceae	Bradyrhizobium	0.67	0.33	223.33	0	0	0
OTU466926611	Bacteria	Proteobacteria	Gammaproteobacteria	Enterobacterial	Enterobacteriaceae	Serratia	48	101.33	2.33	11.33	51.33	8.33
OTU580122866	Bacteria	Proteobacteria	Gammaproteobacteria	Enterobacterial	Enterobacteriaceae	Serratia	71.33	139.33	0.33	1.33	8.33	0.33
OTU197946457	Bacteria	Proteobacteria	Gammaproteobacteria	Enterobacterial	Enterobacteriaceae	Serratia	74.33	133	0.33	1.33	8	1.33
OTU290854999	Bacteria	Proteobacteria	Gammaproteobacteria	Enterobacterial	Enterobacteriaceae	Serratia	65.33	138	0.33	1	4.33	2
OTU506608589	Bacteria	Proteobacteria	Gammaproteobacteria	Enterobacterial	Enterobacteriaceae	Serratia	72	127.33	0	1.33	3.67	0
OTU459147990	Bacteria	Proteobacteria	Gammaproteobacteria	Betaproteobacterial	Burkholderiaceae	Burkholderia-Caballeronia-Paraburkholderia	1	0	200.33	0	0	0
OTU664865098	Bacteria	Proteobacteria	Gammaproteobacteria	Enterobacterial	Enterobacteriaceae	-	98.33	66	1	14.67	0.33	21
OTU950266442	Bacteria	Proteobacteria	Gammaproteobacteria	Enterobacterial	Enterobacteriaceae	Serratia	31.67	165	0	0.33	0.67	0
OTU152265884	Bacteria	Actinobacteria	Actinobacteria	Pseudonocardial	Pseudonocardiaceae	Pseudonocardia	0	0	177.33	0	0	0

OTU314881516	Bacteria	Proteobacteria	Gammaproteobacteria	Enterobacterial	Enterobacteriaceae	Serratia	57	116.67	0	1	1.33	0.33
OTU101793506	Bacteria	Proteobacteria	Alphaproteobacteria	Rhizobiales	Rhizobiaceae	-	19.33	36	0	41.33	61.67	16.33
OTU538188992	Bacteria	Proteobacteria	Gammaproteobacteria	Enterobacterial	Enterobacteriaceae	Serratia	53.67	106	1	1	4.67	1.33
OTU69159101	Bacteria	Proteobacteria	Gammaproteobacteria	Enterobacterial	Enterobacteriaceae	Serratia	51.67	108	0.67	1.67	4.33	0
OTU673071066	Bacteria	Proteobacteria	Gammaproteobacteria	Enterobacterial	Enterobacteriaceae	Escherichia-Shigella	52	95	0.67	5	6	3.33
OTU831900348	Bacteria	Proteobacteria	Alphaproteobacteria	Sphingomonadales	Sphingomonadaceae	Sphingomonas	0	0	159.33	0	0	0
OTU698843918	Bacteria	Proteobacteria	Gammaproteobacteria	Enterobacterial	Enterobacteriaceae	Serratia	9	148.67	0.67	0	0	0
OTU920442650	Bacteria	Proteobacteria	Alphaproteobacteria	Rhizobiales	Rhizobiaceae	Allorhizobium-Neorhizobium-Pararhizobium-Rhizobium	0	1	156	0	0	0
OTU558353147	Bacteria	Proteobacteria	Gammaproteobacteria	Enterobacterial	Enterobacteriaceae	Serratia	54.33	94.67	0.67	1.33	3.33	1
OTU373904307	Bacteria	Proteobacteria	Alphaproteobacteria	Rhizobiales	Rhizobiaceae	-	25.33	36.67	0	31.67	45	15.33
OTU720705245	Bacteria	Proteobacteria	Gammaproteobacteria	Enterobacterial	Enterobacteriaceae	Serratia	47	100	0	1.33	4.33	1.33
OTU207626863	Bacteria	Proteobacteria	Alphaproteobacteria	Rhizobiales	Rhizobiaceae	-	30	41.67	0	25	38.67	16
OTU285816526	Bacteria	Firmicutes	Bacilli	Bacillales	Paenibacillaceae	Paenibacillus	0	99.33	0.33	47	3.67	0

OTU192274036	Bacteria	Proteobacteria	Alphaproteobacteria	Rhizobiales	Rhizobiaceae	Mesorhizobium	1.33	0	57.33	85	6	0
OTU932196013	Bacteria	Proteobacteria	Gammaproteobacteria	Enterobacterial	Enterobacteriaceae	Serratia	51.33	90.33	0.67	1.33	5.33	0
OTU404439014	Bacteria	Bacteroidetes	Bacteroidia	Chitinophagales	Chitinophagaceae	uncultured	0	0	148	0	0	0
OTU539147104	Bacteria	Proteobacteria	Gammaproteobacteria	Betaproteobacterial	Burkholderiaceae	Achromobacter	19	82.33	0	34.67	9.33	0
OTU790778634	Bacteria	Proteobacteria	Alphaproteobacteria	Rhizobiales	Rhizobiaceae	Phyllobacterium	10.67	24.67	0	30.33	33	45
OTU223109408	Bacteria	Proteobacteria	Gammaproteobacteria	Enterobacterial	Enterobacteriaceae	Serratia	49	89.67	0	0.33	4	0
OTU948569439	Bacteria	Proteobacteria	Alphaproteobacteria	Rhizobiales	Rhizobiaceae	Ochrobacrum	13	28.67	0	36.67	43	21
OTU129943922	Bacteria	Proteobacteria	Alphaproteobacteria	Rhizobiales	Rhizobiaceae	-	18.33	32	0	29	43.67	19
OTU739482380	Bacteria	Firmicutes	Bacilli	Bacillales	Bacillaceae	Bacillus	0	0	0	0	140	0
OTU809132203	Bacteria	Firmicutes	Bacilli	Lactobacillus	Carnobacteriaceae	Granulicatella	0	0	0	0	140	0
OTU908122454	Bacteria	Proteobacteria	Gammaproteobacteria	Enterobacterial	Enterobacteriaceae	Serratia	46	85.67	0.33	0.67	4	0.67
OTU440243022	Bacteria	Firmicutes	Bacilli	Bacillales	Staphylococcusaceae	Staphylococcus	0	0	0	135	0	0
OTU263352044	Bacteria	Actinobacteria	Actinobacteria	Micromonosporales	Micromonosporaceae	Actinoplanes	0	0	134.33	0	0	0
OTU705440874	Bacteria	Actinobacteria	Actinobacteria	Corynebacteriales	Nocardiaceae	Rhodococcus	0.33	1	129	3	0.33	0.33
OTU165405924	Bacteria	Proteobacteria	Gammaproteobacteria	Enterobacterial	Enterobacteriaceae	Serratia	43	85.33	0.33	1.67	2.67	1

OTU118180943	Bacteria	Firmicutes	Bacilli	Bacillales	Bacillaceae	Bacillus	0	0	0	0	121	11
OTU9531286	Bacteria	Proteobacteria	Gammaproteobacteria	Enterobacterial	Enterobacteriaceae	Serratia	42	84	0	1.33	2.33	0
OTU197748826	Bacteria	Proteobacteria	Alphaproteobacteria	Rhizobiales	Rhizobiaceae	Mesorhizobium	2.33	0.67	45.67	74.33	4	0
OTU474741974	Bacteria	Proteobacteria	Alphaproteobacteria	Rhizobiales	Rhizobiaceae	-	16	27	0.33	31.67	33.67	17.67
OTU965595576	Bacteria	Proteobacteria	Gammaproteobacteria	Betaproteobacterial	Burkholderiaceae	Achromobacter	1.33	14	1	85.67	20	0
OTU715969667	Bacteria	Proteobacteria	Alphaproteobacteria	Rhizobiales		-	18	28.67	1.67	24.33	35	13.67
OTU893138607	Bacteria	Firmicutes	Bacilli	Bacillales	Bacillaceae	Bacillus	0	0	0	118.33	1.33	0
OTU835814021	Bacteria	Proteobacteria	Gammaproteobacteria	Betaproteobacterial	Burkholderiaceae	Achromobacter	22.33	70	0.33	19.67	5.33	0
OTU5086199	Bacteria	Proteobacteria	Alphaproteobacteria	Rhizobiales	Rhizobiaceae	-	12.67	25.33	0	29	34.67	15.67
OTU550200693	Bacteria	Proteobacteria	Gammaproteobacteria	Enterobacterial	Enterobacteriaceae	Serratia	12	103	0	0	0.67	0
OTU164354793	Bacteria	Proteobacteria	Gammaproteobacteria	Enterobacterial	Enterobacteriaceae	Serratia	32.33	78.67	0	0.33	1.67	0.67
OTU336758870	Bacteria	Proteobacteria	Alphaproteobacteria	Rhizobiales	Rhizobiaceae	-	14	28	0	20	33.33	17
OTU257331586	Bacteria	Firmicutes	Bacilli	Bacillales	Bacillaceae	Bacillus	0	0	0	0	0	111
OTU630391590	Bacteria	Proteobacteria	Alphaproteobacteria	Rhizobiales	Rhizobiaceae	Ensifer	13	27	0.67	22.33	36.33	11
OTU604214932	Bacteria	Proteobacteria	Gammaproteobacteria	Enterobacterial	Enterobacteriaceae	Serratia	35	69.67	0	2	2.67	0.67

OTU417086356	Bacteria	Proteobacteria	Alphaproteobacteria	Rhizobiales	Rhizobiaceae	-	13	21.33	0	26.33	36	13.33
OTU981164454	Bacteria	Proteobacteria	Alphaproteobacteria	Sphingomonadales	Sphingomonadaceae	Sphingomonas	0.33	0	106.67	1.33	0	0
OTU467883596	Bacteria	Firmicutes	Bacilli	Bacillales	Bacillaceae	Bacillus	0	0	0	0	107	0
OTU302723448	Bacteria	Proteobacteria	Gammaproteobacteria	Enterobacterial	Enterobacteriaceae	Pantoea	4.67	101.33	0.33	0	0	0
OTU403024136	Bacteria	Proteobacteria	Alphaproteobacteria	Rhizobiales	Rhizobiaceae	Allorhizobium-Neorhizobium-Pararhizobium-Rhizobium	12.67	22.33	0	21.67	32.33	17.33
OTU89471939	Bacteria	Proteobacteria	Gammaproteobacteria	Betaproteobacterial	Burkholderiaceae	Achromobacter	11.67	49.67	0.67	36	8	0
OTU605416035	Bacteria	Proteobacteria	Gammaproteobacteria	Enterobacterial	Enterobacteriaceae	Serratia	35	66.67	0	0.67	2.33	0.33
OTU623352348	Bacteria	Proteobacteria	Gammaproteobacteria	Enterobacterial	Enterobacteriaceae	Serratia	34	62	0	0.67	3.67	0.67
OTU544544613	Bacteria	Proteobacteria	Gammaproteobacteria	Betaproteobacterial	Burkholderiaceae	Delftia	54.33	32.33	1	9	0.33	0
OTU618418564	Bacteria	Proteobacteria	Gammaproteobacteria	Betaproteobacterial	Burkholderiaceae	Achromobacter	1.33	26	0.67	47.33	16.67	0
OTU347120381	Bacteria	Proteobacteria	Alphaproteobacteria	Rhizobiales	Rhizobiaceae	Allorhizobium-Neorhizobium-Pararhizobium-Rhizobium	10	18.67	0.33	15.67	35	11
OTU572190040	Bacteria	Proteobacteria	Gammaproteobacteria	Enterobacterial	Enterobacteriaceae	-	56	34	0	0	0.33	0
OTU650576483	Bacteria	Proteobacteria	Gammaproteobacteria	Enterobacterial	Enterobacteriaceae	Pectobacterium	2.67	86.33	0	0.67	0	0.33

OTU41286390	Bacteria	Proteobacteria	Gammaproteobacteria	Enterobacterial	Enterobacteriaceae	Serratia	27.33	56.67	0	1.67	2.67	0
OTU27862345	Bacteria	Proteobacteria	Gammaproteobacteria	Enterobacterial	Enterobacteriaceae	Serratia	5.33	82.33	0	0.33	0	0
OTU70851223	Bacteria	Proteobacteria	Alphaproteobacteria	Rhizobiales	Rhizobiaceae	Allorhizobium-Neorhizobium-Pararhizobium-Rhizobium	12	22.67	3	19.33	22	7.33
OTU914465481	Bacteria	Gemmatimonadetes	Gemmatimonadetes	Gemmatimonadales	Gemmatimonadaceae	uncultured	0	0	83.33	0	0	0
OTU499227576	Bacteria	Proteobacteria	Alphaproteobacteria	Micropepsales	Micropepsaceae	uncultured	0	0	83	0	0	0
OTU818719146	Bacteria	Actinobacteria	Actinobacteria	Pseudonocardial	Pseudonocardiaceae	Actinomycetospora	0	0	82.67	0	0	0
OTU493066906	Bacteria	Proteobacteria	Gammaproteobacteria	Enterobacterial	Enterobacteriaceae	Serratia	24.33	55	0	0.33	2.67	0
OTU983052006	Bacteria	Proteobacteria	Alphaproteobacteria	Rhizobiales	Rhizobiaceae	-	24.33	54	0	0.67	1.67	0
OTU612332619	Bacteria	Proteobacteria	Gammaproteobacteria	Enterobacterial	Enterobacteriaceae	Serratia	27.33	35.67	0.33	7.33	8.67	1
OTU87688656	Bacteria	Proteobacteria	Alphaproteobacteria	Rhizobiales	Rhizobiaceae	-	11.67	16	0	21	21	10.67
OTU532160824	Bacteria	Proteobacteria	Alphaproteobacteria	Rhizobiales	Rhizobiaceae	-	8.33	20.33	0	14.67	23.67	10
OTU863246663	Bacteria	Actinobacteria	Actinobacteria	Corynebacteriales	Mycobacteriaceae	Mycobacterium	0	0	76	0	0	0
OTU31929733	Bacteria	Actinobacteria	Actinobacteria	Propionibacterial	Nocardioidaceae	Nocardioids	0	0	75.67	0	0	0

OTU612612684	Bacteria	Firmicutes	Bacilli	Bacillales	Bacillaceae	Bacillus	0	0	0	0	75.33	0
OTU900083652	Bacteria	Firmicutes	Bacilli	Bacillales	Bacillaceae	Bacillus	0	0	0	0	75.33	0
OTU14159098	Bacteria	Proteobacteria	Gammaproteobacteria	Enterobacterial	Enterobacteriaceae	Serratia	20	52.67	0	0.67	1.67	0
OTU433709286	Bacteria	Firmicutes	Bacilli	Bacillales	Sporolactobacillaceae	-	0	0	0	0	3	72
OTU463131479	Bacteria	Proteobacteria	Gammaproteobacteria	Enterobacterial	Enterobacteriaceae	Serratia	18.33	45.67	0.33	0.67	0.33	0
OTU974043033	Bacteria	Proteobacteria	Alphaproteobacteria	Rhizobiales	Rhizobiaceae	Allorhizobium-Neorhizobium-Pararhizobium-Rhizobium	0.33	1	63	0	0.33	0
OTU780899368	Bacteria	Proteobacteria	Gammaproteobacteria	Betaproteobacterial	Burkholderiaceae	Cupriavidus	13.33	48.67	2	0	0	0
OTU172135261	Bacteria	Proteobacteria	Gammaproteobacteria	Enterobacterial	Enterobacteriaceae	Serratia	19.67	40.67	0.67	0.67	2	0.33
OTU360031728	Bacteria	Proteobacteria	Gammaproteobacteria	Salinisphaerales	Solimonadaceae	Nevskya	0	0	63.33	0	0	0
OTU531537210	Bacteria	Proteobacteria	Alphaproteobacteria	Rhizobiales	Rhizobiaceae	-	9.33	15.33	0	12.67	18.67	7
OTU34875128	Bacteria	Proteobacteria	Gammaproteobacteria	Enterobacterial	Enterobacteriaceae	Serratia	17.33	43.33	0	0.67	0.67	0.33
OTU991192808	Bacteria	WPS-2	metagenome	metagenome	metagenome	metagenome	0	0	62.33	0	0	0
OTU40072909	Bacteria	Proteobacteria	Alphaproteobacteria	Rhizobiales	Rhizobiaceae	Ochrobacrum	6.33	14	0	10.67	23.67	7.33
OTU848129002	Bacteria	Proteobacteria	Gammaproteobacteria	Enterobacterial	Enterobacteriaceae	Enterobacter	1.67	57	0.33	0.33	0.67	0

OTU676971859	Bacteria	Proteobacteria	Alphaproteobacteria	Rhizobiales	Rhizobiaceae	-	7	15	2.67	11	18	5.67
OTU997583455	Bacteria	Firmicutes	Bacilli	Bacillales	Bacillaceae	Bacillus	0	0	0	0	59.33	0
OTU259631065	Bacteria	Actinobacteria	Actinobacteria	Micrococcal	Microbacteriaceae	Leucobacter	0	0	0	12.33	17	28.67
OTU151882338	Bacteria	Proteobacteria	Alphaproteobacteria	Rhizobiales	Rhizobiaceae	Ensifer	4.67	9	0	16.33	19.33	6.33
OTU378559456	Bacteria	Proteobacteria	Gammaproteobacteria	Enterobacterial	Enterobacteriaceae	Enterobacter	0.67	32	2.33	5.67	11.33	2.67
OTU611425121	Bacteria	Proteobacteria	Gammaproteobacteria	Enterobacterial	Enterobacteriaceae	Serratia	6.33	20.67	0.33	2.67	19.67	4.33
OTU335410902	Bacteria	Proteobacteria	Gammaproteobacteria	Enterobacterial	Enterobacteriaceae	-	2.33	48.33	0	0.33	1	0.33
OTU586874793	Bacteria	Proteobacteria	Alphaproteobacteria	Rhizobiales	Beijerinckiaceae	-	0	0	52.33	0	0	0
OTU692999591	Bacteria	Cyanobacteria	Oxyphotobacteria	Chloroplast		-	0	0	52	0	0	0
OTU926257942	Bacteria	Actinobacteria	Actinobacteria	Pseudonocardial	Pseudonocardiaceae	Pseudonocardia	0	0	51.33	0	0	0
OTU872920001	Bacteria	Proteobacteria	Gammaproteobacteria	Betaproteobacterial	Burkholderiaceae	Achromobacter	8.67	26.33	0	11	4	0.33
OTU318495066	Bacteria	Proteobacteria	Gammaproteobacteria	Enterobacterial	Enterobacteriaceae	Enterobacter	1.67	42.33	0.67	2.67	2	0.33
OTU932064020	Bacteria	Proteobacteria	Gammaproteobacteria	Enterobacterial	Enterobacteriaceae	Serratia	5	6.33	1.33	8	24	4.67
OTU549787071	Bacteria	Proteobacteria	Gammaproteobacteria	Enterobacterial	Enterobacteriaceae	Serratia	13	34	0	0.33	1.67	0
OTU959216457	Bacteria	Proteobacteria	Gammaproteobacteria	Enterobacterial	Enterobacteriaceae	Serratia	16.33	29.33	0	1	1	0.67

OTU365948501	Bacteria	Proteobacteria	Alphaproteobacteria	Rhizobiales	Beijerinckiaceae	Microvirga	0	0	47.33	0	0	0
OTU936557930	Bacteria	Firmicutes	Bacilli	Bacillales	Bacillaceae	-	0	0	0	0	36.33	11
OTU446154903	Bacteria	Proteobacteria	Alphaproteobacteria	Rhizobiales	Beijerinckiaceae	Methylobacterium	0	0	46	0	0	0
OTU514376659	Bacteria	Proteobacteria	Gammaproteobacteria	Betaproteobacterial	Burkholderiaceae	Achromobacter	6.33	30.67	0	6.67	2.33	0
OTU696929972	Bacteria	Proteobacteria	Gammaproteobacteria	Enterobacterial	Enterobacteriaceae	Serratia	8	22	0	5.67	4.67	5.33
OTU780829089	Bacteria	Proteobacteria	Alphaproteobacteria	Sphingomonadales	Sphingomonadaceae	Sphingomonas	0	0	42	1.33	0	0.33
OTU95129430	Bacteria	Proteobacteria	Gammaproteobacteria	Enterobacterial	Enterobacteriaceae	Serratia	13	28.67	0.33	0	1.67	0
OTU812604762	Bacteria	Proteobacteria	Gammaproteobacteria	Enterobacterial	Enterobacteriaceae	Salmonella	1	42.33	0	0	0	0
OTU399558242	Bacteria	Proteobacteria	Gammaproteobacteria	Enterobacterial	Enterobacteriaceae	Serratia	11.33	29	0	0	0.67	0.33
OTU656473097	Bacteria	Proteobacteria	Gammaproteobacteria	Enterobacterial	Enterobacteriaceae	Enterobacter	0.33	39.67	0	0	0	0
OTU131452829	Bacteria	Proteobacteria	Gammaproteobacteria	Enterobacterial	Enterobacteriaceae	Serratia	15.33	23.67	0	0	0.33	0
OTU200392402	Bacteria	Proteobacteria	Gammaproteobacteria	Enterobacterial	Enterobacteriaceae	Serratia	12.33	24.67	0	0	1.33	0
OTU206946662	Bacteria	Proteobacteria	Gammaproteobacteria	Betaproteobacterial	Burkholderiaceae	Achromobacter	7	23.33	0	6.67	1.33	0
OTU239146798	Bacteria	Proteobacteria	Alphaproteobacteria	Rhizobiales	Rhizobiaceae	-	4.33	9.33	0	3.67	11.33	9.67
OTU746708169	Bacteria	Proteobacteria	Gammaproteobacteria	Enterobacterial	Enterobacteriaceae	Serratia	11.67	25.33	0.33	0	0.33	0

OTU194326192	Bacteria	Proteobacteria	Gammaproteobacteria	Gammaproteobacteria Incertae Sedis	Unknown Family	Acidibacter	0	0	37.33	0	0	0
OTU313399993	Bacteria	Proteobacteria	Gammaproteobacteria	Enterobacterial	Enterobacteriaceae	-	1	34.67	0	1.33	0	0.33
OTU268647399	Bacteria	Actinobacteria	Actinobacteria	Micrococcal	Microbacteriaceae	Microbacterium	0	0	0	35.67	0	1
OTU417431778	Bacteria	Proteobacteria	Alphaproteobacteria	Rhizobiales	Rhizobiaceae	-	3.67	8	0	8.67	10.33	5.33
OTU462008900	Unassigne d					-	5.67	28.33	0	1.33	0	0
OTU775910308	Bacteria	Proteobacteria	Gammaproteobacteria	Enterobacterial	Enterobacteriaceae	-	0.67	33	0.67	0.67	0.33	0
OTU319069836	Bacteria	Proteobacteria	Alphaproteobacteria	Sphingomonadales	Sphingomonadaceae	Sphingomonas	0	0	34.67	0	0	0
OTU926691003	Bacteria	Proteobacteria	Gammaproteobacteria	Betaproteobacterial	Burkholderiaceae	-	10	15.33	5	0.67	3	0
OTU589965645	Bacteria	Proteobacteria	Gammaproteobacteria	Enterobacterial	Enterobacteriaceae	Serratia	10.33	20.33	0	0	2	1
OTU841785969	Bacteria	Proteobacteria	Gammaproteobacteria	Enterobacterial	Enterobacteriaceae	Serratia	11	20.67	0.33	0.67	1	0
OTU886133834	Bacteria	Bacteroidetes	Bacteroidia	Chitinophagales	Chitinophagaceae	uncultured	0	0	32.67	0	0	0
OTU73369040	Bacteria	Proteobacteria	Gammaproteobacteria	Enterobacterial	Enterobacteriaceae	Serratia	21	10.67	0	0.33	0	0
OTU787288677	Bacteria	Firmicutes	Bacilli	Bacillales	Bacillaceae	Bacillus	0	0	0	30.33	0.67	0
OTU375520726	Bacteria	Proteobacteria	Alphaproteobacteria	Sphingomonadales	Sphingomonadaceae	-	0	0	30.33	0.33	0	0

OTU346724469	Bacteria	Proteobacteria	Alphaproteobacteria	Rhizobiales	Rhizobiaceae	Allorhizobium-Neorhizobium-Pararhizobium-Rhizobium	1.67	5.67	15	1	6.33	0.67
OTU183954724	Bacteria	Proteobacteria	Gammaproteobacteria	Betaproteobacterial	Burkholderiaceae	Achromobacter	3	20.67	0.33	4	2.33	0
OTU985022636	Bacteria	Proteobacteria	Gammaproteobacteria	Enterobacterial	Enterobacteriaceae	Serratia	13.33	16.67	0	0	0.33	0
OTU19917900	Bacteria	Actinobacteria	Actinobacteria	Streptosporangiales	Nocardiopsaceae	Nocardiopsis	0	0	0	0	0	29.67
OTU302663086	Bacteria	Proteobacteria	Gammaproteobacteria	Enterobacterial	Enterobacteriaceae	Enterobacter	2	22.33	0.33	2.33	0	2
OTU983758295	Bacteria	Proteobacteria	Gammaproteobacteria	Betaproteobacterial	Burkholderiaceae	Burkholderia-Caballeronia-Paraburkholderia	0	4	24.33	0.33	0	0
OTU1504412	Bacteria	Proteobacteria	Gammaproteobacteria	Enterobacterial	Enterobacteriaceae	Serratia	4.33	23.67	0	0	0.33	0
OTU572543706	Bacteria	Proteobacteria	Gammaproteobacteria	Enterobacterial	Enterobacteriaceae	Serratia	9.67	16.67	0	0	1.67	0
OTU638497225	Bacteria	Proteobacteria	Gammaproteobacteria			-	9.33	18.33	0	0	0	0.33
OTU586628206	Bacteria	Chloroflexi	TK10	uncultured bacterium	uncultured bacterium	uncultured bacterium	0	0	27.67	0	0	0
OTU702154859	Bacteria	Proteobacteria	Gammaproteobacteria	Enterobacterial	Enterobacteriaceae	Enterobacter	2.67	19.33	0	2.67	0.67	1.67
OTU541068589	Bacteria	Proteobacteria	Deltaproteobacteria	Myxococcales	Polyangiaceae	Pajaroellobacter	0	0	26.67	0	0	0
OTU688849265	Bacteria	Proteobacteria	Deltaproteobacteria	Myxococcales	Polyangiaceae	Pajaroellobacter	0.33	0	26.33	0	0	0

OTU647708891	Bacteria	Proteobacteria	Alphaproteobacteria	Caulobacterales	Caulobacteraceae	Brevundimonas	1	0	24.67	0	0.33	0
OTU905355312	Bacteria	Proteobacteria	Alphaproteobacteria	Rhizobiales	Beijerinckiaceae	Methylobacterium	0	0	26	0	0	0
OTU427339448	Bacteria	Actinobacteria	Thermoleophilia	Solirubrobacterales	Solirubrobacteraceae	Patulibcter	0	0	25.33	0	0	0
OTU908001853	Bacteria	Proteobacteria	Alphaproteobacteria	Rhizobiales	Rhizobiaceae	Allorhizobium-Neorhizobium-Pararhizobium-Rhizobium	2	6.33	0	5	9.67	1.33
OTU633716882	Bacteria	Proteobacteria	Gammaproteobacteria	Enterobacterial	Enterobacteriaceae	Serratia	6	17.33	0	0	0	0
OTU687936469	Bacteria	Proteobacteria	Alphaproteobacteria	Rhizobiales	Beijerinckiaceae	Methylobacterium	0	0	22.67	0.33	0	0
OTU699499005	Bacteria	Proteobacteria	Gammaproteobacteria	Betaproteobacterial	Burkholderiaceae	Delftia	8.33	8.33	0	6.33	0	0
OTU989457058	Bacteria	Proteobacteria	Gammaproteobacteria	Betaproteobacterial	Burkholderiaceae	-	10	4	0.67	6.67	1.67	0
OTU252762484	Bacteria	Proteobacteria	Gammaproteobacteria	Betaproteobacterial	Burkholderiaceae	Achromobacter	3.67	13.33	0.33	4.67	0.33	0
OTU424825934	Bacteria	Proteobacteria	Alphaproteobacteria	Rhizobiales	Beijerinckiaceae	Bosea	0	0.33	3	18.67	0	0.33
OTU425908725	Bacteria	Proteobacteria	Gammaproteobacteria	Enterobacterial	Enterobacteriaceae	Serratia	6.67	12.67	0	0.33	0.67	0.67
OTU200863455	Bacteria	Proteobacteria	Gammaproteobacteria	Enterobacterial	Enterobacteriaceae	Serratia	2.33	18	0	0	0	0
OTU248165000	Bacteria	Proteobacteria	Gammaproteobacteria	Enterobacterial	Enterobacteriaceae	Serratia	8.33	11.67	0	0	0.33	0

OTU320064583	Bacteria	Actinobacteria	Thermoleophilia	Solirubrobacterales	67-14	uncultured bacterium	0	0	20.33	0	0	0
OTU399667952	Bacteria	Proteobacteria	Alphaproteobacteria	Rhizobiales	Beijerinckiaceae	Methylobacterium	0	0	20.33	0	0	0
OTU475018487	Bacteria	Actinobacteria	Actinobacteria	Micrococcal	Microbacteriaceae	Leucobacter	0	0	0	9	3.33	7.67
OTU284463876	Bacteria	Proteobacteria	Gammaproteobacteria			-	1.67	6.33	0	3	5.33	2.67
OTU51570595	Bacteria	Proteobacteria	Gammaproteobacteria	Enterobacterial	Enterobacteriaceae	Serratia	5	12.67	0	0	1.33	0
OTU409091255	Bacteria	Proteobacteria	Gammaproteobacteria	Betaproteobacterial	Burkholderiaceae	Delftia	12.33	4.67	0	1.67	0	0
OTU28779889	Bacteria	Firmicutes	Bacilli	Bacillales	Sporolactobacillaceae	Alkalicoccus	0	0	0	0	0	18.33
OTU587247579	Bacteria	Proteobacteria	Gammaproteobacteria	Enterobacterial	Enterobacteriaceae	Serratia	5	13	0	0	0	0
OTU373130543	Bacteria	Actinobacteria	Actinobacteria	Micrococcal	Micrococcaceae	Synomonas	0	0	17.67	0	0	0
OTU915275248	Bacteria	Proteobacteria	Gammaproteobacteria	Enterobacterial	Enterobacteriaceae	Serratia	7	10.33	0	0	0.33	0
OTU854582385	Bacteria	Proteobacteria	Gammaproteobacteria	Enterobacterial	Enterobacteriaceae	Serratia	3	14	0	0	0.33	0.33
OTU931818388	Bacteria	Actinobacteria	Actinobacteria	Corynebacteriales	Mycobacteriaceae	Mycobacterium	0	0	17.33	0	0	0
OTU917589990	Bacteria	Proteobacteria	Gammaproteobacteria	Enterobacterial	Enterobacteriaceae	Serratia	5.33	11.33	0	0	0	0
OTU705647543	Bacteria	Proteobacteria	Gammaproteobacteria	Enterobacterial	Enterobacteriaceae	Serratia	4	11.33	0	0.33	0.67	0

OTU170635592	Bacteria	Proteobacteria	Alphaproteobacteria	Rhizobiales	Rhizobiaceae	Ensifer	2.33	3.67	0	1.33	6.33	2.67
OTU857220458	Bacteria	Proteobacteria	Gammaproteobacteria	Xanthomonadales	Xanthomonadaceae	Stenotrophomonas	0	0	0	15.67	0.67	0
OTU929292250	Bacteria	Firmicutes	Bacilli	Bacillales	Paenibacillaceae	Paenibacillus	0	0	0	0	16.33	0
OTU442436640	Bacteria	Bacteroidetes	Bacteroidia	Chitinophagales	Chitinophagaceae	Segetibacter	14.33	0	0	1.67	0	0
OTU680341304	Bacteria	Proteobacteria	Gammaproteobacteria	Xanthomonadales	Xanthomonadaceae	Stenotrophomonas	0	0.33	0	15	0	0
OTU443765938	Unassigne d					-	5.33	9.67	0	0	0	0
OTU460168031	Bacteria	Proteobacteria	Gammaproteobacteria	Xanthomonadales	Xanthomonadaceae	Stenotrophomonas	0	0	0.67	13	1.33	0
OTU624651850	Bacteria	Bacteroidetes	Bacteroidia	Sphingobacteriales	env.OPS 17	uncultured bacterium	0	0	15	0	0	0
OTU183773416	Bacteria	Proteobacteria	Gammaproteobacteria	Betaproteobacterial	Burkholderiaceae	Achromobacter	1.33	10.33	0	2	0.67	0
OTU266431575	Bacteria	Proteobacteria	Gammaproteobacteria	Enterobacterial	Enterobacteriaceae	Serratia	2.67	11.33	0	0.33	0	0
OTU865836137	Bacteria	Proteobacteria	Gammaproteobacteria	Enterobacterial	Enterobacteriaceae	Serratia	4.33	9.33	0	0	0	0
OTU16012698	Bacteria	Proteobacteria	Alphaproteobacteria	Caulobacterales	Caulobacteraceae	Brevundimonas	0.33	0.33	13	0	0	0
OTU17287945	Bacteria	Proteobacteria	Alphaproteobacteria	Rhizobiales	Beijerinckiaceae	Methylobacterium	0	0	13.67	0	0	0
OTU479360838	Bacteria	Actinobacteria	Actinobacteria	Micrococcal	Microbacteriaceae	Microbacterium	0	0	2.67	5	5.33	0.33

OTU916730666	Bacteria	Proteobacteria	Alphaproteobacteria	Rhizobiales	Rhizobiaceae	Mesorhizobium	0	0	7.33	6	0	0
OTU500432650	Bacteria	Proteobacteria	Alphaproteobacteria	Sphingomonadales	Sphingomonadaceae	Sphingomonas	0	0	13	0	0	0
OTU512691653	Bacteria	Proteobacteria	Gammaproteobacteria	Enterobacterial	Enterobacteriaceae	Enterobacter	0	12.33	0	0	0.33	0
OTU181595787	Bacteria	Proteobacteria	Gammaproteobacteria	Enterobacterial	Enterobacteriaceae	Serratia	3.33	4.33	0	0.33	3.67	1
OTU353547002	Bacteria	Actinobacteria	Actinobacteria	Pseudonocardial	Pseudonocardiaceae	Actinomycetospora	0	0	12.33	0	0	0
OTU919230014	Bacteria	Proteobacteria	Gammaproteobacteria	Betaproteobacterial	Burkholderiaceae	Ralstonia	0	0	12.33	0	0	0
OTU98105994	Bacteria	Actinobacteria	Actinobacteria	Micrococcal	Microbacteriaceae	Leucobacter	0	0	1.33	2.67	5	3
OTU709058444	Bacteria	Proteobacteria	Alphaproteobacteria	Rhizobiales	Rhizobiaceae	-	3.33	5	0	1.67	1.67	0
OTU771323726	Bacteria	Actinobacteria	Actinobacteria	Frankiales	Sporichthyaceae	hgcI clade	5.33	6.33	0	0	0	0
OTU820026000	Bacteria	Proteobacteria	Alphaproteobacteria	Rhizobiales		-	0	0	11.67	0	0	0
OTU239103467	Bacteria	Proteobacteria	Gammaproteobacteria	Enterobacterial	Enterobacteriaceae	-	3	7.67	0	0.33	0	0
OTU281818210	Bacteria	Proteobacteria	Gammaproteobacteria	Enterobacterial	Enterobacteriaceae	Serratia	3.33	7.33	0	0	0.33	0
OTU757906506	Bacteria	Firmicutes	Bacilli	Bacillales	Bacillaceae	Bacillus	0	0	0	0	10.67	0
OTU10527579	Bacteria	Proteobacteria	Alphaproteobacteria	Rhizobiales	Rhizobiaceae	-	5	2	0	0.67	1.67	1

OTU566158490	Bacteria	Proteobacteria	Alphaproteobacteria	Rhizobiales	Rhizobiaceae	-	1	2.33	0	2	3.67	1.33
OTU875441030	Bacteria	Proteobacteria	Gammaproteobacteria	Enterobacterial	Enterobacteriaceae	Citrobacter	0	10.33	0	0	0	0
OTU175511271	Bacteria	Proteobacteria	Gammaproteobacteria	Enterobacterial	Enterobacteriaceae	-	4	5	0	0.67	0	0.33
OTU669664052	Bacteria	Proteobacteria	Alphaproteobacteria	Rhizobiales	Rhizobiaceae	Pseudaminobacter	0	0	0	8.67	1.33	0
OTU859744326	Bacteria	Proteobacteria	Gammaproteobacteria	Altermonadales	Pseudoalteromonadaceae	Pseudoalteromonas	0	0	6.67	0.67	2.67	0
OTU783309517	Bacteria	Proteobacteria	Gammaproteobacteria	Enterobacterial	Enterobacteriaceae	-	5.67	3	0	0.33	0	0.67
OTU176203639	Bacteria	Proteobacteria	Gammaproteobacteria	Enterobacterial	Enterobacteriaceae	Serratia	1.33	1.67	0	2	4	0.67
OTU345844084	Bacteria	Actinobacteria	Actinobacteria	Micrococcal	Micrococcaceae	-	0	0	9.33	0	0	0
OTU430279365	Bacteria	Proteobacteria	Gammaproteobacteria	Betaproteobacterial	Burkholderiaceae	-	0	9.33	0	0	0	0
OTU536092240	Bacteria	Proteobacteria	Gammaproteobacteria	Enterobacterial	Enterobacteriaceae	Serratia	3.67	5.67	0	0	0	0
OTU881889713	Bacteria	Actinobacteria	Actinobacteria	Pseudonocardial	Pseudonocardiaceae	Amycolatopsis	0	0	9.33	0	0	0
OTU417597122	Bacteria	Proteobacteria	Gammaproteobacteria	Enterobacterial	Enterobacteriaceae	Serratia	3	6	0	0	0	0
OTU6639747	Bacteria	Actinobacteria	Actinobacteria	Micrococcal	Micrococcaceae	Pseudarthrobacter	0	0	9	0	0	0
OTU785437128	Bacteria	Actinobacteria	Actinobacteria	Micrococcal	Microbacteriaceae	-	0	0	8.67	0.33	0	0

OTU99632210	Bacteria	Proteobacteria	Gammaproteobacteria	Pseudomonas	Pseudomonadaceae	Pseudomonas	0	8.33	0.67	0	0	0
OTU474455312	Bacteria	Proteobacteria	Alphaproteobacteria	Reyranellales	Reyranellaceae	Reyranella	0	0	8.67	0	0	0
OTU335342420	Bacteria	Elusimicrobial	Elusimicrobial	Lineage IV		-	0	0	8	0	0	0
OTU346654226	Bacteria	Actinobacteria	Actinobacteria	Propionibacterial	Nocardioidaceae	Nocardioids	0	0	8	0	0	0
OTU601854075	Bacteria	Actinobacteria	Actinobacteria	Pseudonocardial	Pseudonocardiaceae	Pseudonocardia	0	0	8	0	0	0
OTU861760241	Bacteria	Proteobacteria	Gammaproteobacteria	Betaproteobacterial	Burkholderiaceae	Delftia	4.67	1.33	0	1.33	0	0
OTU175969503	Bacteria	Actinobacteria	Actinobacteria	Micrococcal	Intrasporangiaceae	-	0	0	7	0	0	0
OTU565772165	Bacteria	Proteobacteria	Gammaproteobacteria	Betaproteobacterial	Burkholderiaceae	Achromobacter	0	6.33	0	0.33	0.33	0
OTU363514828	Bacteria	Proteobacteria	Gammaproteobacteria	Enterobacterial	Enterobacteriaceae	Serratia	1	5.67	0	0	0	0
OTU42435091	Bacteria	Proteobacteria	Gammaproteobacteria	Enterobacterial	Enterobacteriaceae	-	4	2	0	0.33	0	0.33
OTU690221097	Bacteria	Proteobacteria	Alphaproteobacteria	Rhizobiales	Rhizobiaceae	-	0.33	2.33	0	1.67	1.67	0.33
OTU170012696	Bacteria	Proteobacteria	Alphaproteobacteria	Rhizobiales		-	0.67	0.67	1.67	1	1.33	1
OTU178460386	Bacteria	Proteobacteria	Gammaproteobacteria	Betaproteobacterial	Burkholderiaceae	Comamonas	0	0	5.67	0	0.33	0
OTU269048406	Bacteria	Acidobacteria	Acidobacteria	Solibacterales	Solibacteraceae (Subgroup 3)	Candidatus Solibacter	6	0	0	0	0	0

OTU382820588	Bacteria	Actinobacteria	Actinobacteria	Micrococcal	Microbacteriaceae	Leucobacter	0	0	0.33	0.33	0.33	5
OTU4914440	Bacteria	Proteobacteria	Gammaproteobacteria	Betaproteobacterial	Burkholderiaceae	Achromobacter	0.33	5	0	0.67	0	0
OTU520220909	Bacteria	Proteobacteria	Alphaproteobacteria	Rhizobiales	Beijerinckiaceae	Methylobacterium	0	0	6	0	0	0
OTU592843226	Bacteria	Proteobacteria	Gammaproteobacteria	Enterobacterial	Enterobacteriaceae	Serratia	2	4	0	0	0	0
OTU911904000	Bacteria	Firmicutes	Bacilli	Bacillales	Bacillaceae	Bacillus	0	0	0	0	5.33	0.67
OTU45004528	Bacteria	Firmicutes	Bacilli	Bacillales	Bacillaceae	Bacillus	0	0	0	0	5	0.33
OTU520746521	Bacteria	Proteobacteria	Alphaproteobacteria	Rhizobiales	Rhizobiaceae	Ensifer	0	1.33	0	1	2.33	0.67
OTU870966595	Bacteria	Firmicutes	Bacilli	Bacillales	Bacillaceae	Bacillus	0	0	0	5.33	0	0
OTU923098576	Bacteria	Proteobacteria	Alphaproteobacteria	Sneathiellales	Sneathiellaceae	Taonella	0	0	4.67	0.33	0.33	0
OTU118989400	Bacteria	Proteobacteria	Alphaproteobacteria	Rhizobiales	Rhizobiaceae	Allorhizobium-Neorhizobium-Pararhizobium-Rhizobium	0	0	5	0	0	0
OTU399033681	Bacteria	Proteobacteria	Gammaproteobacteria	Betaproteobacterial	Burkholderiaceae	Comamonas	0	0	5	0	0	0
OTU928016440	Bacteria	Proteobacteria	Alphaproteobacteria	Rhizobiales	Beijerinckiaceae	1174-901-12	0	0	0	5	0	0
OTU9760167	Bacteria	Proteobacteria	Gammaproteobacteria	Betaproteobacterial	Burkholderiaceae	Comamonas	1.67	1.67	0.33	1.33	0	0
OTU917764753	Bacteria	Proteobacteria	Gammaproteobacteria	Enterobacterial	Enterobacteriaceae	Serratia	1.33	3.33	0	0	0	0

OTU175218956	Bacteria	Bacteroidetes	Bacteroidia	Bacteroidal	Prolixibacteraceae	Roseimarinus	0	0	3	0	1.33	0.33
OTU345641755	Bacteria	Proteobacteria	Gammaproteobacteria	Betaproteobacterial	Burkholderiaceae	Polynucleobacter	3.67	1	0	0	0	0
OTU730085565	Bacteria	Proteobacteria	Gammaproteobacteria	Enterobacterial	Enterobacteriaceae	Hafnia-Obesumbacterium	1.67	2.67	0	0	0	0.33
OTU991383561	Bacteria	Firmicutes	Bacilli	Bacillales	Bacillaceae	Bacillus	0	0	0	0	4.33	0.33
OTU135522927	Bacteria	Proteobacteria	Gammaproteobacteria	Xanthomonadales	Xanthomonadaceae	Stenotrophomonas	0	0	0.33	4	0	0
OTU401058886	Bacteria	Proteobacteria	Gammaproteobacteria	Betaproteobacterial	Burkholderiaceae	Burkholderia-Caballeronia-Paraburkholderia	0	0	4.33	0	0	0
OTU52940958	Bacteria	Actinobacteria	Actinobacteria	Micrococcal	Micrococcaceae	Arthrobacter	0	0	4.33	0	0	0
OTU550291675	Bacteria	Proteobacteria	Alphaproteobacteria	Sphingomonadales	Sphingomonadaceae	uncultured	0	0	3.67	0.67	0	0
OTU921418729	Bacteria	Proteobacteria	Gammaproteobacteria	Enterobacterial	Enterobacteriaceae	Enterobacter	0	4.33	0	0	0	0
OTU118694879	Bacteria	Patescibacteria	Saccharimonadia	Saccharimonadales	uncultured bacterium	uncultured bacterium	1.67	2.33	0	0	0	0
OTU600558129	Bacteria	Acidobacteria	Acidobacteria	Solibacterales	Solibacteraceae (Subgroup 3)	Bryobacter	4	0	0	0	0	0
OTU79935228	Bacteria	Proteobacteria	Alphaproteobacteria	Rhizobiales	Rhizobiaceae	-	0	0	0	1	1.67	1.33
OTU106274476	Bacteria	Proteobacteria	Gammaproteobacteria	Enterobacterial	Enterobacteriaceae	Escherichia-Shigella	1.67	1.33	0	0.33	0.33	0

OTU256972502	Bacteria	Acidobacteria	Acidobacteria	Solibacterales	Solibacteraceae (Subgroup 3)	Candidatus Solibacter	3.33	0.33	0	0	0	0
OTU492693954	Bacteria	Proteobacteria	Gammaproteobacteria	Enterobacterial	Enterobacteriaceae	Serratia	1.67	2	0	0	0	0
OTU689959385	Bacteria	Proteobacteria	Gammaproteobacteria	Enterobacterial	Enterobacteriaceae	Serratia	2.33	1.33	0	0	0	0
OTU735243278	Bacteria	Proteobacteria	Gammaproteobacteria	Vibrionales	Vibrionaceae	Vibrio	0	0	2	0	1.67	0
OTU786151943	Bacteria	Proteobacteria	Gammaproteobacteria	Enterobacterial	Enterobacteriaceae	Serratia	0.67	3	0	0	0	0
OTU135357500	Bacteria	Proteobacteria	Deltaproteobacteria	Myxococcales	Polyangiaceae	Pajaroellobacter	0	0	3.33	0	0	0
OTU189341722	Bacteria	Proteobacteria	Gammaproteobacteria	Enterobacterial	Enterobacteriaceae	-	2.33	1	0	0	0	0
OTU343984306	Bacteria	Proteobacteria	Gammaproteobacteria	Betaproteobacterial	Burkholderiaceae	Acidovorax	0.33	3	0	0	0	0
OTU498804666	Bacteria	Proteobacteria	Alphaproteobacteria	Caulobacterales	Caulobacteraceae	Brevundimonas	0	0	3.33	0	0	0
OTU574762455	Bacteria	Proteobacteria	Gammaproteobacteria	Betaproteobacterial	Burkholderiaceae	Burkholderia-Caballeronia-Paraburkholderia	0	0	3.33	0	0	0
OTU711886475	Bacteria	Proteobacteria	Gammaproteobacteria	Betaproteobacterial	Burkholderiaceae	Polynucleobacter	1.67	1.67	0	0	0	0
OTU794492313	Bacteria	Acidobacteria	Acidobacteria	Acidobacteriales	uncultured	uncultured bacterium	3	0.33	0	0	0	0
OTU832217965	Bacteria	Proteobacteria	Alphaproteobacteria	Caulobacterales	Caulobacteraceae	Brevundimonas	0	0	3	0.33	0	0

OTU841556246	Bacteria	Proteobacteria	Gammaproteobacteria	Betaproteobacterial	Burkholderiaceae	Delftia	3	0.33	0	0	0	0
OTU87550929	Bacteria	Proteobacteria	Gammaproteobacteria	Enterobacterial	Enterobacteriaceae	Salmonella	0.33	3	0	0	0	0
OTU88675822	Bacteria	Bacteroidetes	Bacteroidia	Chitinophagales	Chitinophagaceae	Asinibacterium	0	0	3.33	0	0	0
OTU951858482	Bacteria	Actinobacteria	Actinobacteria	Frankiales	Frankiaceae	Jatrophihabitans	0	0	3.33	0	0	0
OTU153014457	Bacteria	Proteobacteria	Alphaproteobacteria	Rhizobiales	Rhizobiaceae	Allorhizobium-Neorhizobium-Pararhizobium-Rhizobium	0	1.67	0	1	0.33	0
OTU143966002	Bacteria	Proteobacteria	Gammaproteobacteria	Enterobacterial	Enterobacteriaceae	Serratia	1	2	0	0	0	0
OTU147941245	Bacteria	Proteobacteria	Gammaproteobacteria	Betaproteobacterial	Burkholderiaceae	Comamonas	0	3	0	0	0	0
OTU213353301	Bacteria	Proteobacteria	Gammaproteobacteria	Betaproteobacterial	Burkholderiaceae	Aquabacterium	2.33	0.67	0	0	0	0
OTU281334282	Bacteria	Bacteroidetes	Bacteroidia	Chitinophagales	Chitinophagaceae	Asinibacterium	0	0	3	0	0	0
OTU53527373	Bacteria	Proteobacteria	Gammaproteobacteria	Betaproteobacterial	Burkholderiaceae	-	0	0	3	0	0	0
OTU646978035	Bacteria	Actinobacteria	Actinobacteria	Pseudonocardial	Pseudonocardiaceae	Amycolatopsis	0	0	3	0	0	0
OTU731689269	Bacteria	Proteobacteria	Gammaproteobacteria	Enterobacterial	Enterobacteriaceae	Escherichia-Shigella	0	3	0	0	0	0
OTU929755930	Bacteria	Firmicutes	Bacilli	Bacillales	Bacillaceae	Bacillus	0	0	0	0	3	0

OTU893363143	Bacteria	Proteobacteria	Gammaproteobacteria	Enterobacterial	Enterobacteriaceae	Serratia	1.67	1	0	0	0	0
OTU180674157	Bacteria	Proteobacteria	Gammaproteobacteria	Enterobacterial	Enterobacteriaceae	Serratia	0.67	2	0	0	0	0
OTU327970507	Bacteria	Proteobacteria	Gammaproteobacteria	Enterobacterial	Enterobacteriaceae	Hafnia-Obesumbacterium	1	1.33	0	0	0	0.33
OTU734688156	Bacteria	Proteobacteria	Gammaproteobacteria	Betaproteobacterial	Burkholderiaceae	Achromobacter	0.33	1.67	0	0	0.67	0
OTU855045370	Bacteria	Proteobacteria	Alphaproteobacteria	Rhizobiales	Rhizobiaceae	-	0	1	0	0.33	1.33	0
OTU871017683	Bacteria	Actinobacteria	Actinobacteria	Micrococcal	Micrococcaceae	Arthrobacter	0	0	2.67	0	0	0
OTU895490405	Bacteria	Proteobacteria	Alphaproteobacteria	Rhizobiales	Rhizobiaceae	Ensifer	0.67	0	0	2	0	0
OTU304357944	Bacteria	Proteobacteria	Gammaproteobacteria	Enterobacterial	Enterobacteriaceae	Serratia	0	2.33	0	0	0	0
OTU454974727	Bacteria	Proteobacteria	Alphaproteobacteria	Rhizobiales	Rhizobiaceae	-	0.33	2	0	0	0	0
OTU553942244	Bacteria	Proteobacteria	Alphaproteobacteria	Rhizobiales	Beijerinckiaceae	Methylobacterium	0	0	2.33	0	0	0
OTU564264832	Bacteria	Bacteroidetes	Bacteroidia	Chitinophagales	Chitinophagaceae	-	0	0	2.33	0	0	0
OTU63433428	Bacteria	Proteobacteria	Gammaproteobacteria	Enterobacterial	Enterobacteriaceae	Serratia	0.67	1.33	0	0	0.33	0
OTU647918893	Bacteria	Actinobacteria	Acidimicrobial	uncultured	uncultured bacterium	uncultured bacterium	1.67	0.67	0	0	0	0
OTU686955465	Bacteria	Firmicutes	Bacilli	Bacillales	Bacillaceae	Bacillus	0	0	0	0	2.33	0

OTU688911705	Bacteria	Acidobacteria	Acidobacteria	Solibacterales	Solibacteraceae (Subgroup 3)	Bryobacter	2.33	0	0	0	0	0
OTU724242277	Bacteria	Actinobacteria	Thermoleophilia	Solirubrobacterales	Solirubrobacteraceae	Solirubrobacter	2	0.33	0	0	0	0
OTU733698987	Bacteria	Proteobacteria	Alphaproteobacteria	Acetobacterales	Acetobacteraceae	Rubritepida	0.33	2	0	0	0	0
OTU784595619	Bacteria	Proteobacteria	Gammaproteobacteria	Xanthomonadales	Xanthomonadaceae	Stenotrophomonas	0	0	0	2.33	0	0
OTU953980942	Bacteria	Proteobacteria	Alphaproteobacteria	Rhizobiales	Rhizobiaceae	Ochrobacrum	0	0.67	0	0	1.67	0
OTU953222078	Bacteria	Proteobacteria	Gammaproteobacteria	Enterobacterial	Enterobacteriaceae	Serratia	1	1.33	0	0	0	0
OTU176745745	Bacteria	Proteobacteria	Alphaproteobacteria	Rhizobiales	Rhizobiaceae	-	0	1.33	0	0.67	0	0
OTU218297954	Bacteria	Proteobacteria	Alphaproteobacteria	Rhodobacterales	Rhodobacteraceae	-	0	0	1.33	0.67	0	0
OTU247514331	Bacteria	Proteobacteria	Alphaproteobacteria	Rhizobiales	Rhizobiaceae	-	0	0.67	0	0	1.33	0
OTU266627551	Bacteria	Proteobacteria	Gammaproteobacteria	Xanthomonadales	Xanthomonadaceae	Stenotrophomonas	0	0	0	1.67	0.33	0
OTU328137230	Bacteria	Proteobacteria	Gammaproteobacteria	Betaproteobacterial	Burkholderiaceae	Achromobacter	0	2	0	0	0	0
OTU421440592	Bacteria	Bacteroidetes	Bacteroidia	Sphingobacteriales	Sphingobacteriaceae	Sphingobacterium	0.33	1.67	0	0	0	0
OTU428763268	Bacteria	Firmicutes	Bacilli	Bacillales	Bacillaceae	Bacillus	0	0	0	0	2	0
OTU86662901	Bacteria	Proteobacteria	Gammaproteobacteria	Enterobacterial	Enterobacteriaceae	Serratia	1.33	0.67	0	0	0	0

OTU895722334	Bacteria	Proteobacteria	Gammaproteobacteria	Betaproteobacterial	Burkholderiaceae	Comamonas	0	2	0	0	0	0
OTU356517393	Bacteria	Proteobacteria	Gammaproteobacteria	Betaproteobacterial	Burkholderiaceae	Achromobacter	1.33	0.33	0	0.33	0	0
OTU379940213	Bacteria	Planctomycetes	Planctomycetacia	Isosphaerales	Isosphaeraceae	Singulisphaera	0	0	1.67	0	0	0
OTU779018858	Bacteria	Actinobacteria	Actinobacteria	Corynebacteriales	Mycobacteriaceae	Mycobacterium	0	0	1.67	0	0	0
OTU786438284	Bacteria	Cyanobacteria	Oxyphotobacteria	Nostocales		-	1.67	0	0	0	0	0
OTU8476191	Bacteria	Proteobacteria	Alphaproteobacteria	Sphingomonadales	Sphingomonadaceae	-	0	0	0	1.67	0	0
OTU120184316	Bacteria	Proteobacteria	Gammaproteobacteria	Betaproteobacterial	Burkholderiaceae	Achromobacter	0	1.33	0	0	0.33	0
OTU153873648	Bacteria	Firmicutes	Clostridia	Clostridiales	Lachnospiraceae	-	0	0	1	0	0.67	0
OTU255689758	Unassigne d					-	0.33	1.33	0	0	0	0
OTU262073965	Bacteria	Proteobacteria	Gammaproteobacteria	Betaproteobacterial	Burkholderiaceae	Achromobacter	0.33	1	0	0.33	0	0
OTU360952793	Bacteria	Proteobacteria	Alphaproteobacteria	Rhizobiales	Rhizobiaceae	Allorhizobium-Neorhizobium-Pararhizobium-Rhizobium	0.33	1	0	0.33	0	0
OTU438489378	Bacteria	Proteobacteria	Gammaproteobacteria	Enterobacterial	Enterobacteriaceae	Serratia	0.67	1	0	0	0	0
OTU540814531	Bacteria	Proteobacteria	Alphaproteobacteria	Rhizobiales	Rhizobiaceae	-	0.67	0.67	0	0	0.33	0

OTU609925010	Bacteria	Proteobacteria	Alphaproteobacteria	Caulobacterales	Caulobacteraceae	Phenylobacterium	0	0.67	1	0	0	0
OTU637363327	Bacteria	Proteobacteria	Alphaproteobacteria	Rhizobiales	Rhizobiaceae	-	0	0	0	0.33	1	0.33
OTU828836216	Bacteria	Proteobacteria	Gammaproteobacteria			-	0.33	1	0	0.33	0	0
OTU100452867	Bacteria	Proteobacteria	Alphaproteobacteria	Rhizobiales	Rhizobiaceae	-	0	0	0	0	1.33	0
OTU138896417	Bacteria	Proteobacteria	Gammaproteobacteria	Enterobacterial	Enterobacteriaceae	Serratia	0.67	0.67	0	0	0	0
OTU183486016	Bacteria	Proteobacteria	Gammaproteobacteria	Betaproteobacterial	Burkholderiaceae	Polynucleobacter	1	0.33	0	0	0	0
OTU198120307	Bacteria	Proteobacteria	Gammaproteobacteria	Betaproteobacterial	Burkholderiaceae	Limnohabitans	0.67	0.67	0	0	0	0
OTU211624775	Bacteria	Proteobacteria	Gammaproteobacteria	Enterobacterial	Enterobacteriaceae	Serratia	0.33	1	0	0	0	0
OTU233813548	Bacteria	Proteobacteria	Gammaproteobacteria	Enterobacterial	Enterobacteriaceae	Enterobacter	0	1.33	0	0	0	0
OTU322317441	Bacteria	Proteobacteria	Alphaproteobacteria	Rhizobiales	Rhizobiaceae	-	0	0.67	0	0.33	0.33	0
OTU341751404	Bacteria	Proteobacteria	Gammaproteobacteria	Enterobacterial	Enterobacteriaceae	Enterobacter	0	1.33	0	0	0	0
OTU356402617	Bacteria	Proteobacteria	Alphaproteobacteria	Rhizobiales	Rhizobiaceae	Ensifer	0	0	0	0.67	0.67	0
OTU387425711	Bacteria	Proteobacteria	Gammaproteobacteria	Betaproteobacterial	Burkholderiaceae	Burkholderia-Caballeronia-Paraburkholderia	0	0	1.33	0	0	0
OTU482637424	Bacteria	Proteobacteria	Alphaproteobacteria	Rhizobiales	Rhizobiaceae	-	0	0	0	0.33	0.67	0.33

OTU487740658	Bacteria	WPS-2	uncultured bacterium	uncultured bacterium	uncultured bacterium	uncultured bacterium	1.33	0	0	0	0	0
OTU611140789	Bacteria	Proteobacteria	Gammaproteobacteria	Enterobacterial	Enterobacteriaceae	Serratia	0.33	1	0	0	0	0
OTU663392884	Bacteria	Proteobacteria	Gammaproteobacteria	Xanthomonadales	Xanthomonadaceae	Stenotrophomonas	0	0	0	1	0.33	0
OTU721393959	Bacteria	Proteobacteria	Alphaproteobacteria	Rhizobiales	Rhizobiaceae	Shinella	0	0.33	0	0.33	0	0.67
OTU735069136	Bacteria	Proteobacteria	Gammaproteobacteria	Enterobacterial	Enterobacteriaceae	Serratia	0	1.33	0	0	0	0
OTU954398696	Bacteria	Proteobacteria	Gammaproteobacteria	Betaproteobacterial	Burkholderiaceae	Achromobacter	0	1.33	0	0	0	0
OTU240449448	Bacteria	Proteobacteria	Gammaproteobacteria	Betaproteobacterial	Burkholderiaceae	Delftia	0.33	0.67	0	0	0	0
OTU334639158	Bacteria	Acidobacteria	Acidobacteria	Acidobacteriales	Acidobacteriaceae (Subgroup 1)	-	1	0	0	0	0	0
OTU366348446	Bacteria	Proteobacteria	Alphaproteobacteria	Rhizobiales	Rhizobiaceae	Allorhizobium-Neorhizobium-Pararhizobium-Rhizobium	0	1	0	0	0	0
OTU420757264	Bacteria	Firmicutes	Bacilli	Bacillales	Sporolactobacillaceae	Alkalicoccus	0	0	0	0	0	1
OTU513376149	Bacteria	Proteobacteria	Gammaproteobacteria	Enterobacterial	Enterobacteriaceae	Serratia	1	0	0	0	0	0
OTU52658929	Bacteria	Proteobacteria	Gammaproteobacteria	Enterobacterial	Enterobacteriaceae	Serratia	0.33	0.67	0	0	0	0
OTU703212487	Bacteria	Proteobacteria	Gammaproteobacteria	Enterobacterial	Enterobacteriaceae	Enterobacter	0.33	0.67	0	0	0	0

OTU734874073	Bacteria	Proteobacteria	Gammaproteobacteria	Betaproteobacterial	Burkholderiaceae	Achromobacter	0.33	0.67	0	0	0	0
OTU779637686	Bacteria	Proteobacteria	Gammaproteobacteria	Betaproteobacterial	Burkholderiaceae	Delftia	0.67	0	0	0.33	0	0
OTU868386312	Bacteria	Proteobacteria	Alphaproteobacteria	Rhizobiales	Rhizobiaceae	-	0.33	0.33	0	0	0	0.33
OTU900955565	Bacteria	Proteobacteria	Gammaproteobacteria			-	0	1	0	0	0	0
OTU916712269	Bacteria	Proteobacteria	Alphaproteobacteria			-	0	1	0	0	0	0
OTU109623631	Bacteria	Proteobacteria	Alphaproteobacteria	Rhizobiales	Rhizobiaceae	-	0.67	0	0	0	0	0
OTU195893168	Bacteria	Proteobacteria	Alphaproteobacteria	Caulobacterales	Caulobacteraceae	Phenylobacterium	0	0	0.67	0	0	0
OTU206518877	Bacteria	Proteobacteria	Gammaproteobacteria	Enterobacterial	Enterobacteriaceae	Serratia	0	0.67	0	0	0	0
OTU272417265	Bacteria	Proteobacteria	Gammaproteobacteria	Enterobacterial	Enterobacteriaceae	Serratia	0.67	0	0	0	0	0
OTU348745772	Bacteria	Proteobacteria	Gammaproteobacteria	Betaproteobacterial	Burkholderiaceae	Achromobacter	0	0.67	0	0	0	0
OTU356916402	Bacteria	Proteobacteria	Alphaproteobacteria	Rhizobiales	Rhizobiaceae	Mesorhizobium	0	0	0.33	0.33	0	0
OTU41749083	Unassigned					-	0	0.67	0	0	0	0
OTU45798036	Bacteria	Proteobacteria	Alphaproteobacteria	Rhizobiales	Rhizobiaceae	-	0	0	0	0.33	0.33	0
OTU475186557	Bacteria	Proteobacteria	Alphaproteobacteria	Rhizobiales	Rhizobiales Incertae Sedis	-	0	0	0	0.67	0	0

OTU518833970	Bacteria	Acidobacteria	Acidobacteria	Acidobacteriales	uncultured	uncultured Acidobacteria bacterium	0.67	0	0	0	0	0
OTU581085280	Bacteria	Proteobacteria	Gammaproteobacteria	Enterobacterial	Enterobacteriaceae	Kluyvera	0.67	0	0	0	0	0
OTU605104730	Bacteria	Proteobacteria	Gammaproteobacteria	Betaproteobacterial	Burkholderiaceae	Delftia	0	0	0	0.67	0	0
OTU611147076	Bacteria	Proteobacteria	Gammaproteobacteria	Enterobacterial	Enterobacteriaceae	Serratia	0	0.67	0	0	0	0
OTU620237428	Bacteria	Proteobacteria	Alphaproteobacteria	Rhizobiales	Methylopilaceae	Methylopila	0	0.33	0	0	0.33	0
OTU660447320	Bacteria	Actinobacteria	Acidimicrobial	Microtrichales	uncultured	metagenome	0.67	0	0	0	0	0
OTU70304105	Unassigne d					-	0.67	0	0	0	0	0
OTU71181078	Bacteria	Bacteroidetes	Bacteroidia	Chitinophagales	Chitinophagaceae	uncultured	0	0	0.67	0	0	0
OTU750410846	Bacteria	Elusimicrobial	Lineage IIa			-	0.67	0	0	0	0	0
OTU815419393	Unassigne d					-	0	0	0	0	0	0.67
OTU836699922	Bacteria	Proteobacteria	Alphaproteobacteria	Sphingomonadales	Sphingomonadaceae	Sphingomonas	0	0	0.67	0	0	0
OTU867334813	Bacteria	Planctomycetes	Planctomycetacia	Pyrellulales	Pirellulaceae	Blastopirella	0	0	0.67	0	0	0
OTU893221731	Bacteria	Proteobacteria	Alphaproteobacteria	Rhizobiales	Rhizobiaceae	Ensifer	0	0	0	0.67	0	0

OTU902363946	Bacteria	Proteobacteria	Alphaproteobacteria	Rhizobiales	Rhizobiaceae	-	0	0.67	0	0	0	0
OTU91632150	Bacteria	Proteobacteria	Alphaproteobacteria	Rhizobiales	Beijerinckiaceae	Methylobacterium	0	0	0.67	0	0	0
OTU924445698	Bacteria	Proteobacteria	Alphaproteobacteria	Rhizobiales	Rhizobiaceae	Pseudaminobacter	0	0.67	0	0	0	0
OTU126598510	Bacteria	Cyanobacteria	Oxyphotobacteria	Pseudanabaenales	Pseudanabaenaceae	Pseudanabaena PCC-7429	0	0	0	0	0.33	0
OTU150151038	Bacteria	Actinobacteria	Actinobacteria	Corynebacteriales	Nocardiaceae	Rhodococcus	0	0	0	0	0	0.33
OTU166382971	Bacteria	Proteobacteria	Gammaproteobacteria	Xanthomonadales	Xanthomonadaceae	Silanimones	0	0	0	0.33	0	0
OTU306974137	Bacteria	Proteobacteria	Alphaproteobacteria	Rhizobiales	Rhizobiaceae	-	0	0	0.33	0	0	0
OTU346966735	Bacteria	Proteobacteria	Gammaproteobacteria	Betaproteobacterial	Burkholderiaceae	Comamonas	0	0	0.33	0	0	0
OTU421854418	Bacteria	Proteobacteria	Gammaproteobacteria	Enterobacterial	Enterobacteriaceae	Enterobacter	0	0.33	0	0	0	0
OTU423180801	Bacteria	Proteobacteria	Gammaproteobacteria	Betaproteobacterial	Rhodocyclaceae	Dechlorosome	0	0	0.33	0	0	0
OTU439779140	Bacteria	Proteobacteria	Gammaproteobacteria	Betaproteobacterial	Burkholderiaceae	-	0	0.33	0	0	0	0
OTU559158078	Bacteria	Proteobacteria	Gammaproteobacteria	Enterobacterial	Enterobacteriaceae	Serratia	0	0	0	0	0.33	0
OTU669206085	Bacteria	Firmicutes	Negativicutes	Selenomonadales	Veillonellaceae	Pelosinus	0	0	0.33	0	0	0
OTU742658879	Bacteria	Proteobacteria	Gammaproteobacteria	Enterobacterial	Enterobacteriaceae	Enterobacter	0	0.33	0	0	0	0

OTU851403721	Bacteria	Actinobacteria	Actinobacteria	Micrococcal	Micrococcaceae	Arthrobacter	0	0	0	0.33	0	0
OTU935235753	Bacteria	Proteobacteria	Gammaproteobacteria	Betaproteobacterial	Burkholderiaceae	Burkholderia-Caballeronia-Paraburkholderia	0	0	0.33	0	0	0
OTU936069000	Bacteria	Firmicutes	Negativicutes	Selenomonadales	Veillonellaceae	Pelosinus	0	0	0	0	0.33	0
OTU939084745	Bacteria	Proteobacteria	Gammaproteobacteria	Betaproteobacterial	Burkholderiaceae	Burkholderia-Caballeronia-Paraburkholderia	0	0	0.33	0	0	0
OTU965872607	Bacteria	Proteobacteria	Gammaproteobacteria	Betaproteobacterial	Burkholderiaceae	Comamonas	0	0	0.33	0	0	0
